# Polytriphenylamine Conjugated Microporous Polymers as Versatile Platforms for Tunable Hydrogen Storage

**DOI:** 10.1002/smll.202407292

**Published:** 2024-10-31

**Authors:** John D. Worth, Annela M. Seddon, Valeska P. Ting, Charl F. J. Faul

**Affiliations:** ^1^ Bristol Composites Institute, School of Civil, Aerospace and Mechanical Engineering University of Bristol University Walk Bristol BS8 1TR UK; ^2^ School of Chemistry University of Bristol Cantock's Close Bristol BS8 1TS UK; ^3^ School of Physics, HH Wills Physics Laboratory University of Bristol Tyndall Avenue Bristol BS8 1QU UK; ^4^ College of Engineering, Computing and Cybernetics Australian National University Canberra ACT 2601 Australia

**Keywords:** adsorption, conjugated microporous polymers, hydrogen storage, polymers, renewable energy

## Abstract

Hydrogen (H_2_) as a fuel source presents a promising route toward decarbonization, though challenges in its storage remain significant. This study explores the synthesis and characterization of polytriphenylamine (PTPA) conjugated microporous polymers (CMPs) for H_2_ storage. Utilizing a combination of Buchwald–Hartwig (BH) coupling, the Bristol–Xi'an Jiaotong (BXJ) approach, and variations in monomer reactive site stoichiometry, a polymer with specific surface areas in excess of 1150 m^2^ g^−1^ and micropore volume of 0.47 cm^3^ g^−1^ is developed. H_2_ storage capacities are measured, achieving excess gravimetric uptakes of 1.65 wt.% at 1 bar and 2.51 wt.% at 50 bar and 77 K, with total capacities reaching 4.40 wt.% at 100 bar and 77 K. Net adsorption isotherms reveal advantages to H_2_ storage using PTPA adsorbents over traditional compression up to pressures of 10 bar at 77 K. High mass transfer coefficients of 4.95 min^−1^ indicate a strong material affinity for H_2_. This study highlights the impact of monomer ratio adjustments on the porosity and excess, total, and net H_2_ adsorption capacities of PTPA‐based CMPs, offering insights into the importance of a non‐stoichiometric monomer concentration when developing efficient CMP‐based H_2_ storage materials.

## Graphical abstract

Polytriphenylamine (PTPA) conjugated microporous polymers (CMPs) are synthesized for hydrogen (H_2_) storage, achieving specific surface areas over 1150 m^2^ g^−1^ and micropore volume of 0.47 cm^3^ g^−1^. H_2_ uptake reached 4.40 wt.% at 100 bar and 77 K. Monomer ratio adjustments significantly impacted porosity and adsorption capacities, offering key insights for developing efficient CMP‐based H_2_ storage materials.

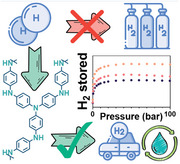

## 1 Introduction

Hydrogen (H_2_) holds great appeal as a promising energy carrier. It is abundantly obtainable from water and biomass sources,^[^ ^1^ ^]^ has the potential to be sustainably produced via carbon‐free methods, and thus is widely anticipated to have a significant impact in shaping a global economy that moves beyond fossil fuels.^[^ ^2^ ^]^ While H_2_ boasts an impressive gravimetric energy density, with a higher heating value three times higher than traditional alternatives^[^ ^3^ ^]^ at ≈140 MJ kg^−1^, its practicality for numerous real‐world applications is hindered by problems with high‐density containment at atmospheric temperatures and pressures.^[^ ^1^, ^4^ ^]^ This poses challenges for its widespread adoption and use.^[^ ^3^ ^]^

The prevailing practice in the H_2_ storage industry to achieve useable densities involves compressing H_2_ gas up to 1000 bar at ambient temperatures,^[^ ^4^ ^]^ before storage in suitable containers. This high‐pressure approach to improving density entails H_2_ gas compression losses. It often necessitates the use of costly, mechanically robust, and lightweight composite materials to ensure the safe operation of the storage system.^[^ ^5^ ^]^ In state‐of‐the‐art Type IV carbon‐fiber‐reinforced high‐pressure vessels, the H_2_ storage capacity per unit mass at 700 bar is approximately equal to 5–7 MJ kg^−1^, significantly lower than the theoretical higher heating value for pure H_2_.^[^ ^6^, ^7^ ^]^ This value accounts for the additional weight of the tank in current compression H_2_ storage system designs. The United States Department of Energy (DoE) has recently established a target for H_2_ storage systems in light‐duty fuel cell vehicles. The ultimate goal (as of 2017) is to achieve an H_2_ storage density of 0.065 kg H_2_ per kg (6.5 wt.%) for onboard storage systems (including all auxiliary equipment).^[^ ^8^ ^]^ Current commercially available mass‐produced passenger vehicles, such as the Toyota Mirai, claim a storage density of ≈5.7 wt.%^[^ ^9^ ^]^ using current compression technology in Type IV tanks at a cost of ≈630 $ kg^−1^ (United States dollar).^[^ ^6^ ^]^

An alternative approach to high‐pressure H_2_ storage is via physisorption.^[^ ^10^ ^]^ This method involves the utilization of porous materials to increase H_2_ density by facilitating interactions between the H_2_ molecules and the internal surfaces of the material. Under comparable conditions of temperature and pressure, it has been observed that this spontaneous adsorption process leads to a higher density of H_2_ (approaching solid‐like phases of 87 kg m^−3^ or greater) compared to more traditional methods, such as compression within tanks.^[^ ^11^, ^12^ ^]^ Furthermore, by reversing the adsorption conditions, such as decreasing pressure or increasing temperature, it becomes possible to fully discharge the stored H_2_ from the material surface. Solid‐state H_2_ storage with porous adsorbents holds the potential to enhance conventional existing storage technologies. Generally, the capacity of a material to physically adsorb H_2_ increases with its surface area, a relationship known as Chahine's rule.^[^ ^13^ ^]^ Materials possessing a large quantity of smaller pores typically have large surface areas, making them good potential adsorbents. In the context of physisorption, it is critical to classify pores according to their size with consistency. The International Union of Pure and Applied Chemistry (IUPAC) recommendation defines micropores as pore diameters not exceeding 2 nm.^[^ ^14^, ^15^ ^]^ Despite the absence of unanimity regarding the ideal pore size for H_2_ storage attributable to variations in adsorbent properties, notable H_2_ uptake capacities are observed in adsorbents featuring micropores spanning from 0.5–0.9 nm^[^ ^16^, ^17^, ^18^ ^]^ owing to enhanced Lennard‐Jones potentials from multiple components of the pore surface.^[^ ^19^ ^]^

Extensive research has been conducted on microporous materials^[^ ^20^, ^21^ ^]^ including zeolites,^[^ ^22^, ^23^ ^]^ activated carbons,^[^ ^24^, ^25^, ^26^ ^]^ carbon nanotubes (CNTs),^[^ ^27^ ^]^ covalent organic frameworks (COFs),^[^ ^28^ ^]^ and metal–organic frameworks (MOFs).^[^ ^29^ ^]^ However, materials with exceedingly high surface areas, such as MOFs, often suffer from inadequate stabilities and pose challenges in terms of synthesis, scale‐up,^[^ ^30^ ^]^ and reproducibility of properties.^[^ ^31^ ^]^ These factors impose limitations on their commercial viability and widespread application.

A class of amorphous organic polymer known as conjugated microporous polymers (CMPs) can exhibit appealing inherent characteristics such as a low skeletal density, chemical and thermal resistance, high microporosity, and large specific surface area (SSA) as determined by nitrogen (N_2_) gas sorption.^[^ ^32^ ^]^ The combination of these properties provides CMPs with certain advantages over other adsorbent materials such as MOFs and COFs, especially in terms of stability.^[^ ^32^, ^33^ ^]^ Additionally, CMPs can be tailored to exhibit distinctive extended π conjugation and a rich heteroatom content throughout their porous 3D networks.^[^ ^32^ ^]^ These properties make CMPs highly suitable for a wide range of applications including gas storage, as their micropores and heteroatom content can significantly boost their affinity for gaseous molecules and lead to increased adsorption capacity.^[^ ^34^ ^]^

The initial CMP characterized for H_2_ storage (and the first CMP to be synthesized), reported in 2007, displayed an SSA of 834 m^2^ g^−1^ and an excess adsorption capacity of approximately 131 cm^3^ g^−1^ (1.10 wt.%) at 77 K and 1.13 bar.^[^ ^35^ ^]^ Subsequent to these findings, advancements in the field of CMP H_2_ storage have resulted in the development of a polycarbazole‐based material exhibiting a measured excess H_2_ capacity of 2.80 wt.% at 77 K and 1 bar.^[^ ^36^ ^]^ This achievement positions CMPs as competitive alternatives to MOFs and activated carbons with greater SSAs.^[^ ^37^, ^38^ ^]^ It is noteworthy that while numerous studies have reported excess H_2_ capacities at 77 K and 1 bar,^[^ ^39^, ^40^, ^41^, ^42^, ^43^, ^44^ ^]^ there is a paucity of investigations assessing the functionality of these materials under elevated pressures.^[^ ^45^, ^46^ ^]^ This aspect warrants consideration, particularly in the context of the potential practical applications of these materials.

Multiple coupling strategies such as Yamamoto,^[^ ^47^, ^48^ ^]^ Suzuki–Miyaura,^[^ ^49^, ^50^ ^]^ and Sonogashira–Hagihara^[^ ^35^ ^]^ have been utilized to synthesize CMPs. The Buchwald–Hartwig (BH) cross‐coupling approach^[^ ^51^, ^52^ ^]^ has also been implemented in CMP design and construction.^[^ ^34^ ^]^ This reaction can be exploited to form carbon–nitrogen bonds between specific monomers in the presence of a palladium catalyst and base to result in polytriphenylamine (PTPA) networks,^[^ ^34^ ^]^ a 3D CMP analogue of the familiar linear redox‐active polymer, polyaniline.^[^ ^53^ ^]^ Subsequent investigations involving these porous networks demonstrated SSAs greater than 1000 m^2^ g^−1^ and excess carbon dioxide (CO_2_) adsorption capacities of 3.60 mmol g^−1^ (16.0 wt.%).^[^ ^54^ ^]^ Until this report, PTPA networks had yet to be explored for H_2_ storage applications.

There have been many investigations into optimizing CMP synthesis strategies to increase SSA. A common approach involves varying the length of the linking organic monomers.^[^ ^39^, ^55^ ^]^ Increasing the number of reactive sites on a participating monomer has also demonstrated improvements to accessible surface area.^[^ ^56^ ^]^ The recently developed Bristol–Xi'an Jiaotong (BXJ) approach^[^ ^54^ ^]^ provides a facile route to unprecedented tuning and enhancement of the porosity of CMPs (formed via various chemistries) by using simple inorganic salts to tune solvent parameters (i.e., Hansen solubility parameters).^[^ ^57^, ^58^ ^]^ Fewer studies exist on the impact of constituent monomer stoichiometry on the impact of properties and functionality of these organic materials. It has been observed that when a stoichiometric ratio of constituent monomers is used in the synthesis of various CMPs, lower porosity polymers are formed than when using a non‐stoichiometric ratio.^[^ ^55^, ^57^ ^]^ Changing the stoichiometric ratios of starting monomers is an underexplored approach to the tuning of physical properties (including SSA) of generated CMPs that can result in enhanced functionality.

In this report, PTPAs constructed from various ratios of feedstock monomers are fully characterized and evaluated for their H_2_ sorption capacities. This investigation aims to provide an understanding of the importance of relative stoichiometry on the porosity of PTPAs and how the material can be tuned for greater porosity and in turn, higher H_2_ adsorption capacities. The estimation of total H_2_ capacity, which is essential for considering the inclusion of porous media in H_2_ storage solutions, is rationalized herein. Net adsorption isotherms are also offered to highlight the advantages of including PTPAs within a potential H_2_ storage system. The materials are compared with other CMP designs reported in the literature under comparable conditions, demonstrating the advantages of this approach.

## 2 Experimental Section

### 2.1 Synthesis of Conjugated Microporous Polytriphenylamines

Tris(4‐bromophenyl)amine, benzene‐1,4‐diamine *(p*‐phenylenediamine), bis(dibenzylideneacetone)palladium(0) (Pd(dba)_2_), 2‐dicyclohexylphosphino‐2′,4′,6′‐triisopropylbiphenyl (XPhos, 97%), sodium *tert*‐butoxide (NaOtBu, 97%), sodium fluoride (NaF) were of analytical research (AR) grade, purchased from Sigma‐Aldrich and used as received. Nuclear magnetic resonance (NMR) and Fourier‐transform infrared (FTIR) spectra of all reagents not shown in the manuscript are offered in Figures S1–S15 (Supporting Information).

A Schlenk tube was charged with the various quantities (see Supporting Information) of tris(4‐bromophenyl)amine and *p*‐phenylenediamine to achieve the desired constituent monomer ratio (and therefore bromine to amine ratio, **Table** **1**), Pd(dba)_2_ (17.25 mg, 0.03 mmol), XPhos (21.45 mg, 0.05 mmol), NaOtBu (336.35 mg, 3.50 mmol), and NaF (20.99 mg, 0.50 mmol). Anhydrous tetrahydrofuran (THF, 30 mL) was added under an N_2_ atmosphere. The reaction mixture was heated to 338 K with stirring under an N_2_ atmosphere for 48 h. The resulting product was collected by centrifugation before being submerged in water (H_2_O), methanol (CH_3_OH), ethanol (C_2_H_6_O), and chloroform (CHCl_3_, 300 mL each) to remove residual catalyst, impurities, and any oligomers. The insoluble polymeric materials were then collected and dried under vacuum (≈10^−3^ bar) at 343 K for a minimum of 24 h.

**Table 1:** Ratio of bromine atoms to amine functional groups in the targeted PTPA CMP material (PTPA‐B_x_N_x_). Yield is reported as a weight percentage.

CMP	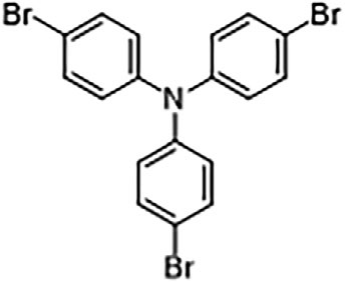 Bromine	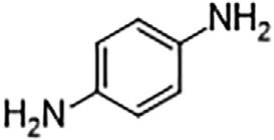 Amine	Yield [%]
PTPA‐Br_1_N_1_	1	1	14
PTPA‐Br_1.5_N_1_	1.5	1	34
PTPA‐Br_2.25_N_1_	2.25	1	40
PTPA‐Br_3_N_1_	3	1	37
PTPA‐Br_1_N_1.5_	1	1.5	12
PTPA‐Br_1_N_2.25_	1	2.25	7
PTPA‐Br_1_N_3_	1	3	< 1

### 2.2 Materials Characterization

#### 2.2.1 Fourier‐Transform Infrared Spectroscopy

To confirm the successful completion of PTPA network formation, FTIR spectra were recorded using a PerkinElmer FTIR Spectrometer Spectrum Two equipped with a lithium tantalate (LiTaO_3_) detector and a diamond crystal in attenuated total reflection (ATR) geometry. An accumulation of four scans was performed in the range of 450–4000 cm^−1^ at a resolution of 4 cm^−1^. The background spectrum was measured separately and then subtracted. A baseline correction was performed on all collected spectra.

#### 2.2.2 Ultraviolet and Visible Near‐Infrared Spectroscopy

Large, highly conjugated systems have characteristic broad wavelength absorption properties. Solid‐state ultraviolet and visible near‐infrared (UV‐Vis‐NIR) spectra were acquired using a Shimadzu UV‐2600 UV‐Vis spectrophotometer equipped with an ISR‐2600Plus two‐detector integrating sphere attachment, covering a wavelength range of 220–1400 nm. A slit width of 5 nm was used. The diffuse reflectance of a sample was initially measured against wavelength before the Kubelka–Munk transformation^[^ ^59^, ^60^ ^]^ was performed to obtain the absorbance spectrum. Prior to data recording, a baseline measurement was conducted using a standard sample composed of barium sulphate (BaSO_4_).

#### 2.2.3 Powder X‐Ray Diffraction

To confirm the amorphous nature of the polymeric materials, powder X‐ray diffraction (PXRD) measurements were conducted using a Bruker D8 Advance instrument equipped with a PSD LynxEye detector and a copper (Cu) K*α* radiation source (*λ* = 1.54 Å). Diffraction patterns were collected in the 2*θ* range of 5–60° with a step size of 0.02° and an exposure time of 1 s step^−1^. Samples were dried and ground to achieve an approximate homogenous particle size before analysis. A silicon sample holder in flat plate geometry was used for each sample. A scan of the empty sample holder was performed as a control and the resulting diffraction pattern is shown in Figure S16 (Supporting Information).

#### 2.2.4 Thermogravimetric Analysis

To obtain information regarding polymer thermal stability (and therefore suitable degassing parameters), thermogravimetric analysis (TGA) was conducted using a NETZSCH STA 449 F1 Jupiter in accordance with criteria published in ISO 11358‐1:2022.^[^ ^61^ ^]^ Approximately 5–10 mg of sample was placed in an alumina crucible (Al_2_O_3_) and heated in an N_2_ atmosphere at a rate of 10 K min^−1^ within the temperature range of 100–850 °C. The pure gas flow rate was 50 mL min^−1^. The protective species flow rate was 20 mL min^−1^. To eliminate any adsorbed moisture content, an isothermal dwell was maintained at 100 °C for 30 min before data collection.

#### 2.2.5 Microscopy

Determination of surface morphology was aided by scanning electron microscopy (SEM). A thin layer of silver (Ag, 99.99% purity), typically 15–20 nm, was coated onto the samples in argon (Ar), to prevent electron charging, using a high‐resolution sputter coater from Agar Scientific. Scanning electron micrographs were obtained on a JEOL JSM‐IT300, operated at an accelerating voltage of 15 kV at a working distance of 10 mm, detecting secondary and backscattered electrons.

#### 2.2.6 Small‐ and Wide‐Angle X‐Ray Scattering

Nanoscale structural features can be determined using small‐ and wide‐angle X‐ray scattering (SAXS and WAXS, respectively). Measurements were performed at ambient temperature under vacuum using a Xenocs SAXSLAB Ganesha 300XL system. The instrument uses a Cu K*α* radiation source (*λ* = 1.54 Å) and a 2D PILATUS X‐ray detector to record the signal before being azimuthally averaged, resulting in scattering intensity (*I*) as a function of the scattering vector length (*Q* = 4πsin*θ*/*λ* ). PTPA samples were loaded into 1.5 mm borosilicate glass capillary tubes (Capillary Tube Supplies Ltd.), and sealed using UV curable adhesive (Norland), and data was collected using two different sample‐detector distances (1050 mm for SAXS and 100 mm for WAXS) and subsequently merged to cover the range *Q* = 0.005–2.6 Å^−1^. Data were collected for an exposure time of 600 s. Data were corrected for transmission and absolute intensity and reduced using SAXSGUI.

#### 2.2.7 Volumetric Nitrogen and Hydrogen Sorption

The porosity and surface characteristics of all samples were assessed using a Micromeritics 3Flex gas sorption analyzer together with N_2_ gas (4.8 grade, 99.998% purity, Air Liquide). Prior to any analysis, 40–80 mg of sample was dried at 345 K in a vacuum oven for a minimum of 24 h. Subsequently, a rigorous in situ degas procedure was performed under high‐vacuum conditions (≈10^−5^ mbar) at 393 K for 12 h to ensure the complete removal of any adsorbed guest molecules from the samples. To avoid any contamination, free/void space analysis of the sample cell was performed using helium (He) gas (99.999% purity, Air Liquide) after N_2_ sorption measurements were completed.

The SSA of all samples was determined using the Brunauer–Emmett–Teller (BET) equation.^[^ ^62^ ^]^ This calculation is based on the linear region of the BET plot, which was obtained from N_2_ isotherms collected at 77 K (maintained with a liquid N_2_ dewar) within the range of relative pressures (*P*/*P*_0_) 10^−7^ ≤ *P*/*P*_0_ ≤ 1, following the criteria established by Rouquerol et al.^[^ ^15^, ^63^ ^]^ for microporous materials and ISO 9277:2022.^[^ ^64^ ^]^ A thermal transpiration correction was applied to the collected isotherms.

The SSA and the specific micropore surface area for all samples were also determined using a 2D non‐local density functional theory (NLDFT) kernel with a slit‐pore model on carbon for N_2_ gas at 77 K.^[^ ^65^ ^]^

Total pore volumes were assessed using both the Gurvitch rule,^[^ ^15^, ^66^ ^]^ which converts the quantity of gas adsorbed into a liquid volume using data points approaching saturation (*P*/*P*_0_ ≈ 0.95), and 2D‐NLDFT.

Differential pore size distribution (PSD) and specific pore volume plots for all samples were realized from the N_2_ isotherms using a 2D‐NLDFT kernel with a slit‐pore model on carbon for N_2_ gas at 77 K.

The evaluation of the H_2_ storage capacity of the materials ≤ 1 bar at 77 K (maintained with a liquid N_2_ dewar) was conducted utilizing a Micromeritics 3Flex gas sorption analyzer in conjunction with H_2_ gas (99.9999% purity, Air Liquide). The volumetric approach was employed, as it is acknowledged for its heightened accuracy at low pressures, attributed to the near‐complete adsorption of the metered dose.^[^ ^67^ ^]^ Prior to any analysis, 40–80 mg of sample was dried at 345 K in a vacuum oven for a minimum of 24 h. Subsequently, a rigorous in situ degas procedure was performed under high‐vacuum conditions (≈10^−5^ mbar) at 393 K for 12 h to ensure the complete removal of any adsorbed guest molecules from the samples. Free/void space analysis of the sample cell was performed using He gas (99.999% purity, Air Liquide) after H_2_ sorption measurements were completed. A thermal transpiration correction was applied to the collected isotherms.

#### 2.2.8 Gravimetric Hydrogen Sorption

To assess the polymer storage capacity in pressurized environments, gravimetric high‐pressure H_2_ gas (99.9999% purity, Air Liquide) sorption analyses were conducted ≤ 100 bar at 77 K using a Hiden Isochema XEMIS‐001 instrument. Prior to measurements, 40–80 mg of dry sample underwent a 12 h in situ degassing process at 393 K under high‐vacuum conditions (≈10^−7^ mbar). A small “plug” of quartz wool of a known density (2.47 g cm^−3^) and mass (≈0.015 g) was positioned above the sample to avoid any loss of polymer under harsh vacuums and gas flow and its volume was considered in all subsequent buoyancy calculations. H_2_ gas equilibration was determined using an asymptotic fit on the sorption‐time curve. The fitting equilibrium relaxation was set to 99% with an allowed fit uncertainty of 5%. The skeletal volume of the samples was determined using He gas pycnometry (99.999% purity, Air Liquide), allowing for the consideration of buoyancy effects at elevated gas densities.

### 2.3 Estimation of Total Adsorption and Net Adsorption

Within the context of research on H_2_ storage materials, a pivotal consideration involves the conversion of Gibbs surface excess adsorption (*n*_ex_) to total adsorption (*n*_tot_). Total adsorption denotes the total quantity of adsorbate within the adsorbed phase and is defined by Myers and Monson^[^ ^67^ ^]^ as,

(1) $$n_{\mathrm{tot}}=n_{\mathrm{ex}}+\rho_{g}V_{p}$$

where *n*_ex_ is the experimentally determined excess adsorption amount, *ρ*_g_ is the molar density of the gas phase at a given temperature and pressure and *V*_p_ is the pore volume of the adsorbent. At low pressures, *ρ*_g_ *V*_p_ is small enough to be considered insignificant; however, this assumption is no longer true at elevated pressures. It must be noted that this commonly adopted equation is only valid for materials comprising purely of micropores.^[^ ^67^, ^68^ ^]^ An illustration to help differentiate between the excess adsorption and total adsorption on an imaginary Gibbs dividing surface is provided in Figure S17 (Supporting Information). It is worth noting that Equation 1 is sometimes given as a description of the absolute adsorption amount (*n*_abs_). *n*_abs_ cannot be directly measured due to the experimental impossibility of precisely determining the position of the Gibbs dividing surface or the dimensions of the adsorbed region. However, for microporous materials, *n*_abs_ and *n*_tot_ can be considered approximately equal values.^[^ ^69^ ^]^

Amorphous materials, such as CMPs, typically exhibit a range of pore sizes that cannot be precisely determined using crystallographic techniques. Gas adsorption offers an estimation, but its accuracy is subject to limitations and may vary based on the specific technique employed. For example, NLDFT calculations assume idealized pore shapes (cylindrical, slit‐shaped, or spherical) and that pore walls are chemically and structurally homogenous. Real materials often have more complex and irregular pore structures. In the case of disordered micro‐ and mesoporous materials, it is inappropriate to assume that every molecule entering the pore network becomes a constituent of the adsorbed phase.^[^ ^68^, ^70^ ^]^

The main alternative approach is to assume that the adsorbed phase has a constant density as a function of concentration and that the adsorbate is not excluded from any pore space until the pore is filled. Such behavior is expected during mesopore filling but is considered less applicable as the pore size decreases. Such an approach postulates a constant density of the adsorbed phase with respect to concentration and concurrently assumes that the adsorbate is not precluded from any pore space until the pore reaches its full capacity. While this behavior aligns with expectations during mesopore filling, its applicability diminishes as pore size decreases. With these assumptions, *n*_tot_ can be defined as,

(2) $$n_{\mathrm{tot}}=\frac{n_{\mathrm{ex}}}{\left(1-\rho_{g}/\rho_{\mathrm{ads}}\right)}\;$$

where *ρ*_ads_ is the adsorbed phase density. The assumption that the density of the adsorbed phase equals that of the liquid phase is a frequently employed approach and is used in this report (*ρ*_ads_  =  0.07085 g cm^−3^ for H_2_). Diagrams illustrating these two assumptions (Equation 1 and Equation 2) can be located in Figures S18 and S19 (Supporting Information). Total adsorption values were calculated using Isochema HIsorb 2019 v4.02.0137. The software uses appropriate data from the NIST Reference Fluid Thermodynamic and Transport Properties Database (REFPROP)^[^ ^71^ ^]^ which is in turn calculated from the Leachman equation‐of‐state for H_2_.^[^ ^72^ ^]^

Net adsorption (*n*_net_) is defined as the differential quantity of gas present in a storage system with an adsorbent compared to without it.^[^ ^73^ ^]^ This metric provides a more accurate evaluation of the advantages of utilizing an adsorbent in H_2_ storage solutions as opposed to conventional compression vessels devoid of adsorbents.^[^ ^68^ ^]^ Consequently, net adsorption is frequently termed the “engineering capacity”. *n*_net_ can be expressed as,

(3) $$n_{\mathrm{net}}=n_{\mathrm{ex}}\;-\left(\rho_{g}\times V_{\mathrm{sk}}\right)$$

where *V*_sk_ is the adsorbent skeletal volume. Again, appropriate data from NIST REFPROP was used for *ρ*_g_ for H_2_ at 77 K between 0 and 100 bar. An isothermal plot of *ρ*_g_ versus pressure (at 77 K) is shown in Figure S20 (Supporting Information).

### 2.4 Hydrogen Adsorption Kinetic Analysis

Real‐time H_2_ gas uptake, pressure, and temperature of the material were recorded while collecting gravimetric H_2_ isotherms. The kinetic data (H_2_ uptake as a function of time) was analyzed to determine the H_2_ adsorption mass transfer coefficient (*k*) using the linear driving force (LDF) model. The LDF for adsorption is described by Glueckauf^[^ ^74^ ^]^ as,

(4) $$\frac{n_{t-t_{1}}-n_{1}}{n_{2}-n_{1}}=1-e^{k\left(t-t_{1}\right)}$$

where the boundary conditions can be described as $n_{t-t_{1}}=n_{1}$ at *t*  = *t*_1_ yielding $\frac{n_{t-t_{1}}-n_{1}}{n_{2}-n_{1}}=0$ and $n_{t-t_{1}}=n_{2}$ at *t* → ∞ yielding $\frac{n_{t-t_{1}}-\;n_{1}}{n_{2}-\;n_{1}}=1$. Here, *n* is the adsorbed amount of H_2_, *t* is time, *n*_1_ is the adsorbed amount at time *t*  = *t*_1_, *n*_2_ is the equilibrium amount of H_2_, and $n_{t-t_{1}}$ is the amount adsorbed at time *t* − *t*_1_.

## 3 Results and Discussion

### 3.1 Synthesis Confirmation and Material Characterization

In this study, a series of materials were synthesized (entries 1–7, Table 1) through a BH coupling reaction involving tris(4‐bromophenyl)amine and *p*‐phenylenediamine as monomers (see Scheme S1 in the Supporting Information). The stoichiometry of these reactants was systematically adjusted to control the ratio of bromine to amine groups in the idealized final structure. The polymers are named with the convention PTPA‐B_x_N_x_ where B_x_N_x_ is the ratio of bromine atoms to amine functional groups present in the targeted structure of the resulting material (expanded detail with an example calculation is given in the Supporting Information, Section 6.1). Typical reactions yielded insoluble blue‐black polymeric materials (see Figure S21, Supporting Information). The formation of soluble black oligomeric species was also noted, as the reaction mixtures remained dark blue‐black, even after the reactions were completed and stirring stopped.

**Figure 1:**
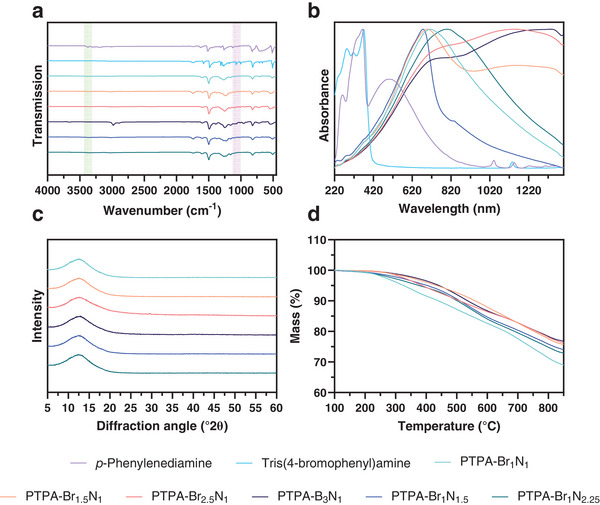
a) Normalized FTIR spectra of monomers and synthesized PTPAs. –NH stretching signal present in monomer and absent in PTPAs as shown by the green band. C–Br stretching signal present in monomer and absent in PTPAs as shown by the pink band. Spectra have been shifted equidistantly on the ***y***‐axis for clarity. b) Normalized UV‐Vis‐NIR absorbance spectra of monomers and synthesized PTPA samples. c) XRD patterns of synthesized amorphous PTPA samples. Patterns have been shifted equidistantly on the ***y***‐axis for clarity. The broad peak visible in all diffraction patterns at 2*θ* ≈ 12° originates from the weakly diffracting amorphous Si sample holder. d) Thermogravimetric curves of synthesized PTPAs obtained under N_2_ atmosphere between 100–850 °C.

It should be noted that the conditions used in the synthesis of PTPA‐Br_1_N_3_ yielded negligible (< 1%) insoluble polymeric material and meaningful characterization was unfeasible. The culminating reaction mixture consisted of dark blue‐black soluble oligomers that remained in solution, owing to the extreme excess of amine to bromine functional groups. The series of the six successful transformations were confirmed by FTIR, UV‐Vis, XRD, and TGA as shown in **Figure** **1**a–d.

The sharp peak present at 1075 cm^−1^ in the FTIR of tris(4‐bromophenyl)amine characteristic of aryl bromide (C–Br) stretching is absent or altered in the FTIR spectra of the polymeric material (Figure 1a).^[^ ^75^ ^]^ Additionally, the absence of any –NH stretching signals (3375 cm^−1^) from the starting monomer shows the transformation of the primary amine moiety to form the respective PTPA entries.^[^ ^34^ ^]^ All resulting materials exhibit quinoid and benzenoid peaks at ≈1595 and ≈1495 cm^−1^, respectively.^[^ ^34^ ^]^ Furthermore, aryl C–H transmission peaks at ≈820 cm^−1^ are also present as expected.^[^ ^34^ ^]^

The UV‐Vis‐NIR spectroscopy reveals significant changes in absorption behavior, characterized by bathochromic shifts and broader absorption profiles as the material transitions from monomer to polymer. These alterations indicate a substantial increase in conjugation, confirming the successful polymerization and formation of the expected CMPs (Figure 1b). The broad absorbance peaks are typical of highly conjugated structures^[^ ^76^ ^]^ and can be attributed to π–π* transitions within the extended, cross‐linked ring system.^[^ ^34^ ^]^ The measured diffuse reflection spectra is shown in Figure S22 (Supporting Information).

Powder XRD measurements (Figure 1c) confirmed the amorphous nature of all synthesized polymers and showed no discernible peaks. Due to the extremely weak diffracting properties of the polymers, a broad peak at 2*θ* ≈ 12° is observable, owing to the presence of the amorphous silicon (Si) sample holder.

Thermogravimetric studies indicated the high thermal stability of PTPA‐Br_1.5_N_1_ and PTPA‐Br_3_N_1_, with onset decomposition temperatures (*T*_o_) exceeding 400 °C (see Figure 1d; Table S1, Supporting Information). In contrast, PTPA‐Br_1_N_1_, exhibited a lower *T*_o_ value of 254 °C, which may be attributed to more limited cross‐linking within the 3D polymeric structure.

**Figure 2:**
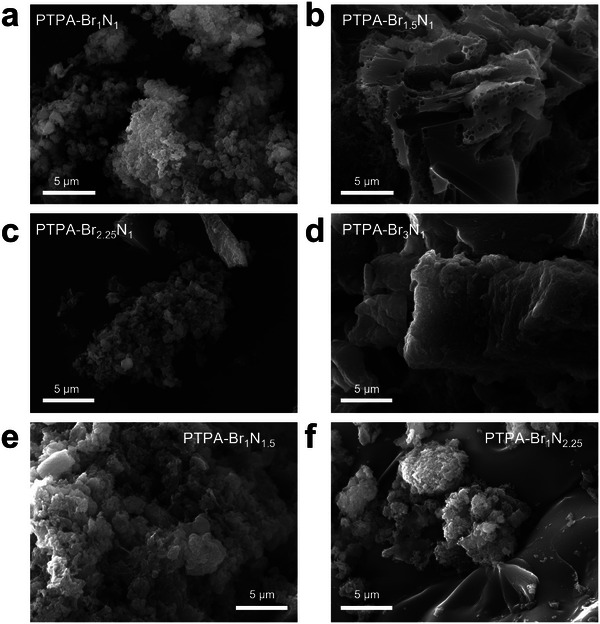
a–f) SEM images of the resulting polymers rendered from detected secondary electrons. Scale bars of 5 µm are included for reference. Sample names are given within the subfigure image.

SEM images revealed the morphology of the PTPAs, confirming the presence of aggregated or fused particulates (**Figure** **2**). Polymers PTPA‐Br_1_N_1_, PTPA‐Br_1_N_1.5_, and PTPA‐Br_1_N_2.25_ appear similar and consist of aggregated nanoparticles that lead to external surface areas and indicate mesoporosity. In contrast, PTPA‐Br_1.5_N_1_ (Figure 2b) exhibits multiple voids with diameters of ≈0.45 µm within the polymeric surface at this magnification.

**Figure 3:**
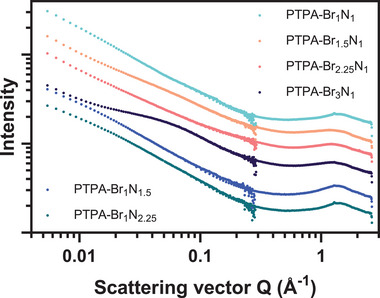
Merged SAXS and WAXS profiles of the PTPA polymeric materials collected in the *Q* range of 0.0005–0.29 Å^−1^ and 0.05–2.64 Å^−1^, respectively. Profiles have been shifted equidistantly on the ***y***‐axis for clarity.

The merged SAXS and WAXS curves shown in **Figure** **3** are comprised of discernible characteristic contributions in the small‐angle regime (*Q* < 1 Å^−1^). In the low *Q* regime (*Q *< 0.02 Å^−1^), the scattering intensity from all samples is dominated by surface scattering from the rough polymeric surfaces (see Figure 2). PTPA‐Br_3_N_1_ exhibits characteristic nanopore scattering for scattering vectors 0.02 < *Q* < 0.1 Å^−1^. Such pattern features have been observed in highly porous activated carbons with narrow pore size distributions.^[^ ^25^, ^77^ ^]^ This characteristic curve shape is not apparent in other samples due to the overlap of SAXS signal because of broader distributions of pore sizes. Such overlap of the signal prevents meaningful evaluation of the SAXS pattern by established models such as the commonly used Guinier approximation.^[^ ^78^ ^]^

The power law can provide information on the structure of a material and describes the relationship between *I* and *Q* as follows,

(5) $$I\left(Q\right)\cdot Q^{-n}$$

where the exponent (also known as the fractal dimension), *n*, is 4 for perfectly smooth surfaces and is lower for rough or diffuse surfaces.^[^ ^79^, ^80^ ^]^ A greater *n* value in Equation 5 suggests that the scattering objects are more compact or dense in structure, leading to a more rapid decrease in *I* as *Q* increases. A lower *n* value indicates more extended, diffuse, or porous structures, resulting in a slower decrease in scattered intensity.^[^ ^81^ ^]^ PTPA‐Br_1.5_N_1_ exhibits a low fractal dimension term of *n* ≈ 3.0, further supporting the morphology alluded to by SEM investigations. In contrast, PTPA‐Br_1_N_1_ has a greater exponent of *n* ≈ 3.5, suggesting a close‐packed and compact structure. It is crucial to note that the power law term for PTPA‐Br_3_N_1_ (*n* ≈ 3.3) may be influenced by the defining curve in the pattern. This feature is characteristic for amorphous adsorbents with narrow pore size distributions. A comparison of the magnitude of *n* for all PTPA samples is compiled in Table S2 (Supporting Information) and the linear regression analysis is shown in Figure S23 (Supporting Information).

The broad shoulder in the WAXS pattern at *Q* > 1 Å^−1^ visible in all samples resulting from diffuse scattering indicates highly disordered, amorphous solid structures.^[^ ^82^, ^83^ ^]^ The width of the peak at half its maximum intensity offers insights into the degree of disorder within the material. A broader peak typically indicates greater amorphous character. This suggests that PTPA‐Br_1.5_N_1_, PTPA‐Br_2.25_N_1_, and PTPA‐Br_3_N_1_ possess a greater number of random, interconnected internal surfaces compared to PTPA‐Br_1_N_1_, PTPA‐Br_1_N_1.5_, and PTPA‐Br_1_N_2.25_.

### 3.2 Surface Area and Porosity Characterization

**Figure 4:**
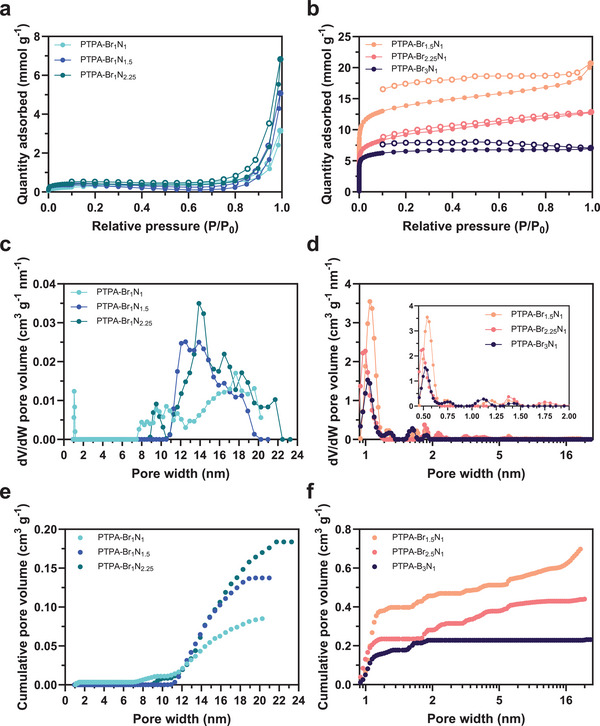
a) N_2_ sorption isotherms collected at 77 K of PTPA‐Br_1_N_1_, PTPA‐Br_1_N_1.5_, and PTPA‐Br_1_N_2.25_. Adsorption branches are denoted by the filled symbol (–•–) and desorption branches by the empty symbol (–◦–). b) N_2_ sorption isotherms collected at 77 K of PTPA‐Br_1.5_N_1_, PTPA‐Br_2.25_N_1_, and PTPA‐Br_3_N_1_. Adsorption branches are denoted by the filled symbol (–•–) and desorption branches by the empty symbol (–◦–). c) Differential pore volume versus pore width derived using a 2D‐NLDFT carbon slit‐pore model for N_2_ adsorption at 77 K for PTPA‐Br_1_N_1_, PTPA‐Br_1_N_1.5_, and PTPA‐Br_1_N_2.25_. d) Differential pore volume versus pore width displayed on a logarithmic scale derived using a 2D‐NLDFT carbon slit‐pore model for N_2_ adsorption at 77 K for PTPA‐Br_1.5_N_1_, PTPA‐Br_2.25_N_1_, and PTPA‐Br_3_N_1_. The inset shows the microporous region only. Cumulative pore volume versus pore width derived using a 2D‐NLDFT carbon slit‐pore model for N_2_ adsorption at 77 K for PTPA‐Br_1_N_1_, PTPA‐Br_1_N_1.5_, and PTPA‐Br_1_N_2.25_. f) Cumulative pore volume versus pore width derived using a 2D‐NLDFT carbon slit‐pore model for N_2_ adsorption at 77 K for PTPA‐Br_1.5_N_1_, PTPA‐Br_2.25_N_1_, and PTPA‐Br_3_N_1_.

Gas sorption studies involving N_2_ reveal significant differences in porosity properties among the materials. According to the classifications by IUPAC,^[^ ^15^ ^]^ PTPA‐Br_1_N_1_, PTPA‐Br_1_N_1.5_, and PTPA‐Br_1_N_2.25_ (**Figure** **4**a) display a Type II isotherm, indicating weak adsorbent–adsorbate interactions typically observed in solids with limited porosity. The linear section of the plot beginning at *P*/*P*_0_ ≈ 0.1 indicates the completion of monolayer coverage. The thickness of the adsorbed multilayer appears to increase indefinitely as *P*/*P*_0_ → 1. Their PSD plots reveal a negligible or non‐existent level of micropore presence, predominantly featuring pore diameters within the mesoporous range. It is noteworthy that the formation of a 3D network from a stoichiometric ratio of aromatic halogen and amino groups leads to a polymer with a reduced surface area (PTPA‐Br_1_N_1_, **Table** **2**), in contrast to samples exhibiting non‐stoichiometric, higher bromine atom concentrations as discussed below.

**Table 2:** Summary of porosity characteristics from N_2_ gas adsorption at 77 K including specific surface areas determined classically (*S*_BET_) and using 2D‐NLDFT (*S*_2D‐NLDFT_), specific micropore surface area (*µS*_2D‐NLDFT_), total pore volumes determined classically (*V*_p_) and using 2D‐NLDFT (*V*_2D‐NLDFT_), and micropore volume (*µV*_p_) determined by 2D‐NLDFT.

CMP	*S*_BET_ [m^2^ g^−1^] ^a)^	*S*_2D‐NLDFT_ [m^2 ^g^−1^] ^b)^	*µS*_2D‐NLDFT_ [m^2^ g^−1^] ^c)^	*V*_p_ [cm^3^ g^−1^] ^d)^	*V*_2D−NLDTF_ [cm^3^ g^−1^] ^e)^	*µV*_p_ [cm^3^ g^−1^] ^f)^
PTPA−Br_1_N_1_	24	18	6	0.04	0.09	0.01
PTPA‐Br_1.5_N_1_	1153	1561	1500	0.64	0.70	0.47
PTPA‐Br_2.25_N_1_	736	1067	1011	0.44	0.44	0.31
PTPA‐Br_3_N_1_	562	720	719	0.25	0.23	0.23
PTPA‐Br_1_N_1.5_	34	19	0	0.06	0.14	0.00
PTPA‐Br_1_N_2.25_	37	24	0	0.08	0.18	0.00

^a)^ Calculated from N_2_ adsorption isotherms collected at 77 K using the BET method;

^b)^ Calculated from N_2_ adsorption isotherms collected at 77 K using 2D‐NLDFT;

^c)^ Cumulative pore surface area in pore sizes ≤ 2 nm calculated from N_2_ adsorption isotherms collected at 77 K using 2D‐NLDFT;

^d)^ Calculated from single point adsorption at *P*/*P*_0_ ≈ 0.95;

^e)^ Calculated from N_2_ adsorption isotherms collected at 77 K using 2D‐NLDFT;

^f)^ Cumulative pore volume in pore sizes ≤ 2 nm calculated from N_2_ adsorption isotherms collected at 77 K using 2D‐NLDFT.

In Figure 4b, PTPA‐Br_1.5_N_1_ and PTPA‐Br_2.25_N_1_ both display a distinctive stepwise increase in the quantity of gas adsorbed at lower *P*/*P*_0_ ranges, which is characteristic of microporous solids. At these lower *P*/*P*_0_ values, PTPA‐Br_1.5_N_1_ and PTPA‐Br_2.25_N_1_ exhibit a Type I(b) isotherm, indicating the presence of a pore size distribution encompassing a range of micropore diameters, resulting in a significant micropore volume. However, at *P*/*P*_0_ ≈ 0.15, the adsorption behavior transitions toward a more Type IV nature owing to the presence of mesopores within the material, allowing for unrestricted monolayer–multilayer adsorption.

The isotherm of PTPA‐Br_3_N_1_ is also depicted in Figure 4b and shows a Type I(b) profile. This isotherm is characterized by an initial rapid uptake of N_2_ gas at lower relative pressures, followed by a discernible shoulder at *P*/*P*_0_ ≈ 0.5, much like in the more porous PTPA‐Br_1.5_N_1_ and PTPA‐Br_2.25_N_1_ polymers. The observed phenomenon is ascribed to the diverse PSD within the microporous range present within the polymeric structure, as illustrated in the PSD plot (Figure 4d). However, as *P*/*P*_0_ exceeds 0.5, there is a notable reduction in additional gas adsorption. This reduction can be attributed to the absence of significant mesopores (> 2 nm) within the material, which would otherwise contribute to continued gas adsorption. The ratio of functional groups in the monomers used to construct PTPA‐Br_3_N_1_ resulted in a purely microporous polymer with negligible mesopore presence, highlighting the tunability of these materials using this approach. This is strongly supported by the aforementioned curve feature in the SAXS pattern (Figure 3). The adsorption branches of the three most porous materials are plotted using a logarithmic *P*/*P*_0_ scale to highlight the micropore filling at low relative pressures in Figure S24 (Supporting Information).

The shapes of hysteresis loops that can manifest in N_2_ isotherms have also been categorized by IUPAC.^[^ ^15^ ^]^ The origin of the Type H4 sorption hysteresis, commonly observed in micro‐meso disordered materials with poorly defined pore shapes and observable in PTPA‐Br_1.5_N_1_ and (to a lesser extent) PTPA‐Br_3_N_1_ can potentially be explained through network models.^[^ ^84^, ^85^, ^86^, ^87^ ^]^ These models take into account that pores can be interconnected, forming a 3D network, and consider pore blockage effects during evaporation of the adsorbate. Such phenomenon occurs when a pore can only connect to the external gas phase through narrow constrictions, often described as ink‐bottle‐shaped pores. In such pores, empty central regions become saturated at elevated relative pressures. However, the emptying of this central region during desorption depends on the initial depletion of the narrow neck of the pore at lower relative pressures. As a result, within a network of ink‐bottle pores, the capillary condensate in these pores is hindered by the presence of liquid in their necks, leading to the desorption branch of the isotherm exhibiting a percolation transition. PTPA‐Br_2.25_N_1_ exhibits minimal hysteresis and less complex pore geometry may be inferred. The collected, unmodified N_2_ sorption data for each polymer are provided electronically as adsorption information files (AIF).^[^ ^88^ ^]^

The analysis of all collected isotherms using the 2D‐NLDFT method with a carbon slit‐pore model for N_2_ adsorption at 77 K revealed the differential (Figure 4c,d) and cumulative (Figure 4e,f) pore size distributions of the materials, respectively. The differential PSD plots of the less porous polymers PTPA‐Br_1_N_1_, PTPA‐Br_1_N_1.5_, and PTPA‐Br_1_N_2.25_ exhibit broad peaks within the mesoporous region (Figure 4c) with only PTPA‐Br_1_N_1_ displaying extremely limited microporosity. In contrast, the PSD plots of the higher bromine content polymers (Figure 4d) display rich microporosity with sharp peaks at 0.45–0.65 nm.

Table 2 summarizes the specific surface areas as determined via the BET method (*S*_BET_) and 2D‐NLDFT (*S*_DFT_), as well as the specific micropore surface area (*µS*_2D‐NLDFT_) calculated via 2D‐NLDFT. The total pore volumes, determined by both classical methods (*V*_p_) and 2D‐NLDFT (*V*_2D‐NLDFT_), along with the micropore volume (*µV*_p_), are also provided. The multipoint BET plots used to calculate *S*_BET_ can be found in Figure S25 (Supporting Information), with a summary of all parameters derived from the fittings listed in Table S3 (Supporting Information).

The porosity characterization indicates that an elevated bromine functionality, up to a certain threshold, contributes to an optimal combination of crosslinking and bridging short sections of linear polymeric links, resulting in the formation of a highly porous structure in PTPA‐Br_1.5_N_1_. Beyond this optimal value, an increased bromine content leads to the formation of polymers with notable reductions in SSA, total pore and micropore volumes, resulting in the less porous structures PTPA‐Br_2.25_N_1_ and PTPA‐Br_3_N_1_, respectively. Polymers PTPA‐Br_1_N_1_, PTPA‐Br_1_N_1.5_, and PTPA‐Br_1_N_2.25_ show similar *S*_BET_ and *S*_DFT_ values, total pore volumes and insignificant or non‐existent micropore volumes.

### 3.3 Hydrogen Sorption

**Figure 5:**
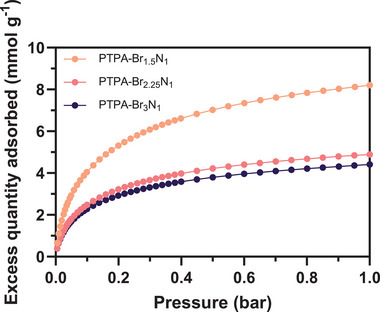
H_2_ adsorption isotherms collected at 77 K at pressures ≤ 1 bar for selected PTPA‐Br_1.5_N_1_, PTPA‐Br_2.25_N_1_, and PTPA‐Br_3_N_1_. The median isotherm from three collected data sets is shown. Only adsorption branches, denoted by the filled symbol (–•–), are shown for clarity. Repeat isotherms are offered in Figures S26–S28 (Supporting Information).

After determining the surface characteristics and porosity of the polymers, their H_2_ uptake capacities were analyzed. At low pressures (≤ 1 bar at 77 K), PTPA‐Br_1.5_N_1_ exhibited the highest volumetrically determined adsorbed excess H_2_ amount at 8.2 mmol g^−1^ (1.65 wt.%, see **Figure** **5** and **Table** **3**). As it is known that BET SSA correlates well with H_2_ uptake at 77 K,^[^ ^21^ ^]^ this outcome was to be expected. The polymer PTPA‐Br_2.25_N_1_ has an excess H_2_ capacity of 4.9 mmol g^−1^ (0.99 wt.%) and PTPA‐Br_3_N_1_ a capacity of 4.3 mmol g^−1^ (0.87 wt.%), both following the same correlation. Interestingly, PTPA‐Br_1.5_N_1_ outperforms other reported CMPs with greater reported BET SSAs (characterized at the same conditions).^[^ ^89^, ^90^ ^]^ For example, NPOF‐1 and NPOF‐2 exhibit BET SSAs of 1907 and 3127 m^2^ g^−1^, respectively with reported H_2_ uptake of < 1.50 wt.% for both polymers. This capability can be attributed to the presence of micropores in PTPA‐Br_1.5_N_1_ (0.46 cm^3^ g^−1^, Table 2) with diameters optimized through variation of the co‐monomer stoichiometry, thus enhancing, and facilitating interactions with H_2_ molecules. At these sites, an enhanced overlap of electrostatic potentials between opposing pore walls creates stronger binding sites, which, in turn, enables a greater enthalpy of adsorption and H_2_ densification. The incorporation of heteroatoms in PTPA‐Br_1.5_N_1_ may also be contributing to a higher isosteric enthalpy of adsorption. Research employing machine learning and focusing on key structural parameters influencing enhanced H_2_ adsorption in activated carbon materials found that improvements in the ultra‐micropore volume (usually defined as pores with a width ≤ 0.7 nm) prove more efficacious than increasing the micropore volume (i.e., pores with a width ≤ 2 nm).^[^ ^91^ ^]^ This appears true within PTPAs as H_2_ storage decreases with a reduction in ultra‐micropore volume (Figure 4d) in PTPA‐Br_1.5_N_1_ (0.38 cm^3^ g^−1^), PTPA‐Br_2.25_N_1_ (0.24 cm^3^ g^−1^), and PTPA‐Br_3_N_1_ (0.16 cm^3^ g^−1^), respectively.

**Table 3:** Volumetrically determined excess H_2_ adsorption capacities (mean of three data sets with standard deviation) for PTPA‐Br_1.5_N_1_, PTPA‐Br_2.25_N_1_, and PTPA‐Br_3_N_1_ at 1 bar and 77 K.

CMP	Excess H_2_ capacity [mmol g^−1^]	Excess H_2_ capacity [wt.%]
PTPA‐Br_1.5_N_1_	8.2 ± 0.1	1.65 ± 0.02
PTPA‐Br_2.25_N_1_	4.9 ± 0.1	0.99 ± 0.02
PTPA‐Br_3_N_1_	4.3 ± 0.2	0.87 ± 0.05

The significantly higher enthalpy of adsorption in PTPA‐Br_1.5_N_1_, in comparison with PTPA‐Br_2.25_N_1_ and PTPA‐Br_3_N_1_, is empirically suggested by the steeper initial H_2_ volumetric uptake observed within the lower pressure region, as shown in Figure 5. The full reversibility and cyclability of each material when analyzed using volumetric techniques is demonstrated in Figure S26–S28. Notably, no hysteresis loop is observed on the desorption branch with H_2_ as the adsorbate, underscoring the suitability as a rechargeable storage medium. The unaltered, volumetrically measured H_2_ gas sorption data for each polymer are provided electronically as AIF files.^[^ ^88^ ^]^

**Figure 6:**
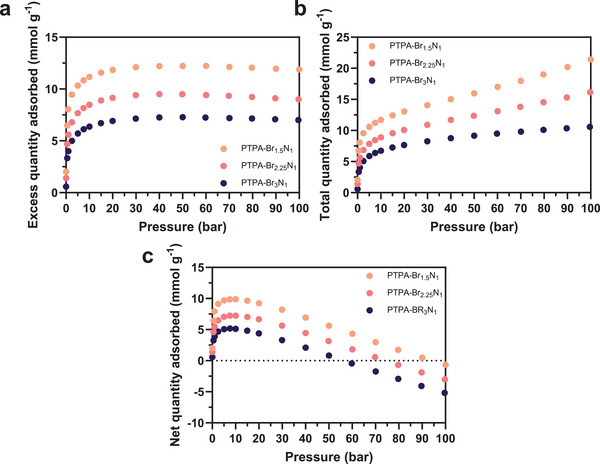
a) Gravimetrically determined H_2_ excess adsorption isotherms collected at 77 K and pressures ≤100 bar for PTPA‐Br_1.5_N_1_, PTPA‐Br_2.25_N_1_, and PTPA‐Br_3_N_1_; b) Calculated total adsorption isotherms at 77 K and pressures ≤ 100 bar for PTPA‐Br_1.5_N_1_, PTPA‐Br_2.25_N_1_, and PTPA‐Br_3_N_1_; c) Calculated net adsorption isotherms at 77 K and pressures ≤ 100 bar for PTPA‐Br_1.5_N_1_, PTPA‐Br_2.25_N_1_, and PTPA‐Br_3_N_1_. Only adsorption branches, denoted by the filled symbol (*•*), are shown for clarity. Repeat isotherms are offered in the Supporting Information (Figures S29–S37, Supporting Information).

To evaluate the suitability for realistic application of the more porous polymeric materials, it is essential to determine the uptake capacities of materials under high‐pressure conditions. Excess H_2_ isotherms were recorded gravimetrically between 0 and 100 bar at 77 K as shown in **Figure** **6**a. The isotherms follow Langmuir‐type behavior,^[^ ^92^ ^]^ with all PTPAs showing a steep increase in adsorbed H_2_ at pressure ≤ 5 bar owing to the presence of sub‐nanometer pores that function as strong adsorption sites. This uptake gradually transitions into a more moderate increase at > 5 bar as the polymer micropore volume becomes fully occupied, leaving (in the case of PTPA‐Br_1.5_N_1_ and PTPA‐Br_2.25_N_1_) only mesoporous space available for further adsorption, which contributes little to storage capability under these conditions.^[^ ^93^ ^]^ Maximum excess uptakes of 12.4, 9.4, and 7.7 mmol g^−1^ were achieved at approximately 50 bar for PTPA‐Br_1.5_N_1_, PTPA‐Br_2.25_N_1_, and PTPA‐Br_3_N_1_, respectively (**Table** **4**). For pressures exceeding the maximum excess adsorption condition, the isotherms show a negative gradient as bulk H_2_ densification occurs.^[^ ^70^, ^94^ ^]^

**Table 4:** Summary of excess, total, and net H_2_ adsorption capacities of PTPA‐Br_1.5_N_1_, PTPA‐Br_2.25_N_1_, and PTPA‐Br_3_N_1_ at 77 K. The skeletal densities (***ρ***_**sk**_) of the polymers are also given.

CMP	Excess H_2_ capacity at 50 bar^a)^		Total H_2_ capacity at 100 bar^b)^		Net H_2_ capacity at 10 bar^c)^		*ρ*_sk_ [g cm^−3^]^d)^
	mmol g^−1^	wt.%	mmol g^−1^	wt.%	mmol g^−1^	wt.%	
PTPA‐Br_1.5_N_1_	12.4 ± 0.2	2.51 ± 0.03	21.8 ± 0.4	4.40 ± 0.07	10.1 ± 0.1	2.03 ± 0.02	1.24 ± 0.03
PTPA‐Br_2.25_N_1_	9.4 ± 0.3	1.89 ± 0.06	15.9 ± 0.6	3.21 ± 0.19	7.2 ± 0.3	1.44 ± 0.06	1.29 ± 0.02
PTPA‐Br_3_N_1_	7.7 ± 0.4	1.56 ± 0.07	11.1 ± 0.4	2.24 ± 0.08	5.5 ± 0.3	1.11 ± 0.06	1.27 ± 0.01

^a)^ Maximum gravimetrically determined excess H_2_ adsorption (mean of repeat measurements with standard deviation);

^b)^ Maximum total H_2_ adsorption calculated with Equation 1 for PTPA‐Br_3_N_1_ and Equation 2 for PTPA‐Br_1.5_N_1_ and PTPA‐Br_2.25_N_1_ (mean of repeat calculations with standard deviation);

^c)^ Maximum net H_2_ adsorption calculated with Equation 3 (mean of repeat calculations with standard deviation);

^d)^ Skeletal density calculated from sample mass and skeletal volume obtained via He gas pycnometry. Uncertainties were calculated from linear regressions fitted to CMP buoyancy versus He gas density plots (Figure S38, Supporting Information).

Three independently collected isotherms, conducted under identical high‐pressure conditions (100 bar, 77 K), are provided. The standard deviation of these results (Table 4) was minimal (e.g., 0.2 mmol g^−1^), indicating consistent performance. This confirms that the material retains its functionality even after multiple exposures to these harsh conditions. Repeat excess isotherms, including desorption branches, are presented in Figures S29–S31 (Supporting Information).

The excess uptake is defined as the amount of H_2_ physically adsorbed within the volume of the sample, with the subtraction of the bulk H_2_ quantity that resides within the structure of the pores.^[^ ^68^, ^70^, ^92^ ^]^ The total quantity of molecules in the adsorbed phase (or the total adsorption) – an important consideration for complete storage systems – can be approximated using the determined excess adsorption data.^[^ ^69^ ^]^ The PTPAs corresponding total adsorption isotherms are shown in Figure 6b and given in Table 4. The total quantity adsorbed amounts for PTPA‐Br_1.5_N_1_ and PTPA‐Br_2.25_N_1_ at 77 K for pressures ≤ 100 bar (21.8 and 15.9 mmol g^−1^, respectively) were derived using Equation 2, owing to their pore size distributions featuring a broad range of diameters. The total adsorbed amount for PTPA‐Br_3_N_1_ at the same conditions (11.1 mmol g^−1^) was estimated using Equation 1 (where *µV*_p_ ≈ *V*_p_ = 0.23 cm^3^ g^−1^ as determined using 2D‐NLDFT) due to considerable evidence that this polymer variant is entirely microporous. Repeat total isotherms with desorption branches present are offered in Figures S32–S34 (Supporting Information). It is reiterated that all total adsorbed quantities are assumptions.

Net H_2_ adsorption (Figure 6c) for each of the PTPAs was calculated using Equation 3 and the determined excess H_2_ adsorption quantities. In the context of gas storage applications, this metric demonstrates the advantages provided by the presence of the adsorbent within the storage solution. The net adsorption for the PTPAs follows a trend similar to excess adsorption, initially increasing to a peak for each material at ≈10 bar, before subsequently decreasing. When the gradient of the plot for each material becomes negative with increasing pressure, the H_2_ storage advantage offered by the material diminishes as the physical volume is occupied by the material, which could otherwise be used to densify H_2_ gas through compression. The point at which net adsorption crosses the pressure axis (***x***‐axis) represents the pressure at which the presence of the adsorbent in the tank no longer provides any benefit at 77 K (≈100, 80, and 60 bar for PTPA‐Br_1.5_N_1,_ PTPA‐Br_2.25_N_1_, and PTPA‐Br_3_N_1,_ respectively). Repeat net isotherms are offered in Figures S35–S37 (Supporting Information).

The skeletal densities (*ρ*_sk_) of the polymers, as calculated by the relationship between sample mass and sample volume (*V*_sk_, Table S4, Supporting Information) as determined via He pycnometry (Figure S38, Supporting Information) and used in the buoyancy correction calculation when gravimetrically determining the Gibbs surface excess amount of H_2_ adsorbed, are also given. These values are not only of critical importance in gravimetric H_2_ adsorption studies but also highlight the low density of the PTPA frameworks, a feature desirable for useful H_2_ storage applications. Well‐studied MOFs such as Cr‐MIL‐100 and UiO‐67 have reported *ρ*_sk_ values of 2.07 and 1.94 g cm^−3^, respectively, indicating more tightly packed structures or higher concentrations of heavier components.^[^ ^95^ ^]^ The *ρ*_sk_ of amorphous carbons derived from biomass sources can vary from 1.37 to 2.08 g cm^−3^ depending on the activation method.^[^ ^96^ ^]^ Consistent with other evidence, PTPA‐Br_1.5_N_1_ exhibits the lowest density (1.24 g cm^−3^). PTPA‐Br_3_N_1_ is less dense when compared with PTPA‐Br_2.25_N_1_ (see Table 4) but is more limited as an H_2_ storage medium by its lower micropore volume. A visualization of these data is given in Figure S39 (Supporting Information).

An important aspect that has received limited attention within the class of CMP adsorbents pertains to the kinetics of H_2_ adsorption, which correlates with the duration required for the charging process of gas within the porous storage system. The H_2_ mass transfer coefficient at constant temperature (77 K) has been assessed in the three highest‐performing PTPAs to provide a quantitative evaluation of the H_2_ adsorption kinetics within the materials. The *k* value has been derived using the LDF model (Equation 4) at the pressure dosing step approaching 1 bar (where H_2_ adsorption in the micropores is still occurring) for each polymer and the pressure dosing step approaching 20 bar (where the polymers start to approach excess adsorption limitations). The greater the value of *k*, the more efficient the diffusion of H_2_ through the material.^[^ ^97^ ^]^ Values of *k* for each material are compiled in **Table** **5**. Plots of the fractional transient uptakes and pressure as a function of time at 77 K for both isothermal steps for all materials are shown in Figures S40–S42 (Supporting Information), including fittings of the LDF that allow for *k* to be calculated.

**Table 5:** Mass transfer coefficient values for PTPA samples determined by the LDF when analyzing kinetic data between two selected isothermal H_2_ dosing steps (approaching 1 bar & approaching 20 bar). Uncertainties calculated from the LDF fittings are given.

CMP	*k* [min^−1^] ^a)^	*k* [min^−1^] ^b)^
PTPA‐Br_1.5_N_1_	4.95 ± 0.08	4.62 ± 0.23
PTPA‐Br_2.25_N_1_	3.36 ± 0.04	3.07 ± 0.01
PTPA‐Br_3_N_1_	3.26 ± 0.05	2.87 ± 0.02

^a)^ Determined using the LDF by analyzing the dosing step between 0.5 and 1 bar;

^b)^ Determined using the LDF by analyzing the dosing step between 15 and 20 bar.

During the lower pressure H_2_ dosing step (0.5–1 bar), the *k* value of the PTPAs exhibits a similar trend as the SSA, micropore volume, and excess H_2_ capacity, with PTPA‐Br_1.5_N_1_ demonstrating the highest performance, characterized by a *k* value of 4.95 min⁻¹. Such an outcome is anticipated since a larger SSA corresponds to a greater number of sites available for H_2_ molecule interaction with the material. The increased number of suitable adsorption sites facilitates more efficient mass transfer by providing more opportunities for molecule adsorption onto the polymer. Materials with higher SSA typically possess more complex surface structures and greater surface heterogeneity. Kinetic analysis has been similarly performed on other H_2_ adsorbents. The activated carbons AX21 and A30 have *k* values of ≈6.5 and ≈4.5 min^−1^ at comparable pressure dosing steps.^[^ ^97^ ^]^ Similarly, the MOF MIL‐101 exhibits a mass transfer coefficient of ≈6.5 min^−1^, measured at analogous pressure steps.^[^ ^97^ ^]^ The processable, organic, linear porous polymer PIM‐1 (in membrane form)^[^ ^98^ ^]^ shows a much lower mass transfer coefficient than the PTPAs with a *k* value of ≈0.6 min^−1^ at similar conditions indicating a lower number of readily accessible appropriate adsorption sites when compared with the aforementioned adsorbents.^[^ ^97^ ^]^ A visualization of these data is given in Figure S43 (Supporting Information).

At a greater pressure dosing step (approaching 20 bar), the mass transfer coefficients for all PTPAs reduces to 4.62, 3.07, and 2.87 min^−1^ for PTPA‐Br_1.5_N_1_, PTPA‐Br_2.25_N_1_, and PTPA‐Br_3_N_1_, respectively. At this isothermal step, the materials are approaching their maximum excess H_2_ capacity, highlighting that micropore filling occurs more rapidly until the volume becomes occupied. Less suitable adsorption sites (mesopores, for example) become occupied at much lower rates.

**Figure 7:**
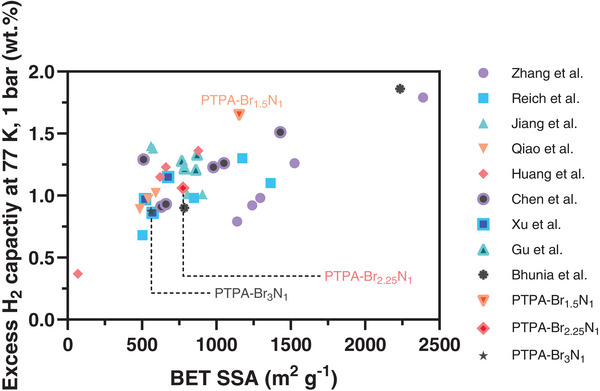
Plot of excess H_2_ capacity (wt.%) of various CMPs at 77 K and 1 bar determined volumetrically as a function of BET SSA (m^2^ g^−1^). Other examples shown are samples from series created with systematic approaches with aims to increase H_2_ adsorption. References: Zhang et al.,^[^ ^101^ ^]^ Reich et al.,^[^ ^90^ ^]^ Jiang et al.,^[^ ^55^ ^]^ Qiao et al.,^[^ ^100^ ^]^ Huang et al.,^[^ ^39^ ^]^ Chen et al.,^[^ ^102^ ^]^ Xu et al.,^[^ ^40^ ^]^ Gu et al.,^[^ ^41^ ^]^ and Bhunia et al.^[^ ^99^ ^]^

A comparison of the performance of PTPAs (under conditions of 77 K and 1 bar) with other CMPs (which were designed, synthesized, and characterized as part of a series) discussed in the literature is presented in **Figure** **7**. Bhunia and co‐workers improved their triazine‐based organic frameworks (PCTF‐1 and PCTF‐2) by altering the concentration of the Lewis acid involved in the synthesis to achieve SSAs of 2235 and 784 m^2^ g^−1^, respectively. The authors report excess H_2_ adsorption capacities of 1.86 and 0.90 wt.% for the polymers.^[^ ^99^ ^]^ Qiao et al. show a relationship between the performance of CMPs concerning H_2_ adsorption and the length of the synthesized rigid skeleton, ultimately concluding that shorter building blocks are preferable. The authors ultimately obtain a maximum excess H_2_ adsorption of 1.02 wt.%, with TEPO‐3 possessing an SSA of 592 m^2^ g^−1^.^[^ ^100^ ^]^ Zhang et al. synthesized a series of covalent triazine‐based frameworks (CTF) by a microwave‐enhanced high‐temperature ionothermal polymerization method, varying time and power, and achieved a maximum SSA of 2390 m^2^ g^−1^ with a reported H_2_ uptake of 17.8 mg g^−1^ (1.78 wt.%).^[^ ^101^ ^]^ Aside from this one high‐performing example, the other prepared CTF samples possess SSAs greater than the majority of CMPs presented in Figure 7 while displaying average performance in H_2_ adsorption, and weaker enthalpies of adsorption.

Importantly, PTPA‐Br_1.5_N_1_ demonstrates higher excess H_2_ adsorption capacities at these conditions when compared with other CMP examples with higher SSAs (Chen et al.,^[^ ^102^ ^]^ Reich et al.,^[^ ^90^ ^]^ and Zhang et al.).^[^ ^101^ ^]^ This advantage may be attributed to its optimized pore diameters and/or an abundance of heteroatoms within its 3D polymeric structure promoting a higher enthalpy of adsorption. It is worth noting that PTPAs can be synthesized under mild conditions at 338 K, whereas the CMPs that surpass the performance of PTPA‐Br_1.5_N_1_ at these conditions require more complex synthesis methods involving microwave radiation^[^ ^101^ ^]^ or heating at 673 K.^[^ ^99^ ^]^

Outside the CMP class of adsorbents, the performance and adsorbate pressures used for characterization vary significantly. The well‐studied MOF, MOF‐5, exhibits a gravimetric excess H_2_ capacity of 1.30 wt.% at 1 bar and 77 K.^[^ ^103^ ^]^ In contrast, NU‐100 has reported maximum gravimetric excess H_2_ capacities of 9.95 wt.% at 77 K and 56 bar.^[^ ^104^ ^]^ Research on single‐walled CNTs indicates that these materials perform predictably according to Chahine's rule, as is typical for carbonaceous materials at cryogenic temperatures.^[^ ^105^ ^]^ Activated carbons, such as the benchmarked commercial TE7, exhibit reported excess gravimetric H_2_ capacities of approximately ≈2.10 wt.%.^[^ ^106^ ^]^ Zeolites, despite decades of research, have demonstrated maximum excess H_2_ gravimetric capacities of only ≈2.20 wt.%.^[^ ^105^, ^107^ ^]^

## 4 Conclusion

The current challenges of gaseous H₂ storage may be overcome through adsorption and H_2_ densification, but new materials are needed to address these issues effectively. In this investigation, a range of tunable, stable, amorphous PTPA networks were designed for H_2_ storage applications, gaining valuable insights into the tuning of CMP porosity through stoichiometric variations in monomer reactive groups. The choice of monomer ratios, with a higher concentration of bromine functionality relative to amine functionality, was crucial in optimizing the structure and porosity of the 3D polymeric structures formed using BH chemistry. This strategic approach produced highly microporous polymers with competitive specific surface areas compared to other CMP designs. SAXS patterns and the corresponding calculated fractal dimensions correlated well with N_2_ gas sorption data and subsequently generated pore size distribution plots.

Elementary kinetic studies of H_2_ adsorption using the LDF model on the PTPA samples highlighted the critical role of porosity and surface characteristics of CMPs in enhancing gas diffusion and mass transfer within the system. The highest‐performing PTPA exhibited mass transfer coefficients comparable to those of activated carbons and significantly greater than other organic polymeric adsorbents, underscoring their suitability for short charging times in practical applications.

These findings position PTPAs as promising candidates for H_2_ storage, outperforming many existing CMPs in the literature in terms of gravimetric capacities. The simplicity and efficiency of the low‐temperature synthetic procedure further enhances the appeal of PTPAs as viable alternatives for efficient H_2_ storage materials compared to CNTs and MOFs.

This study contributes to the understanding of porosity control in PTPA networks and demonstrates their high performance in enhancing H_2_ gravimetric storage capacities, which could reduce the operating pressures of current compression technologies without compromising H_2_ density. The net adsorption isotherms show significant gains in storage ability at lower pressures and 77 K when using PTPAs as H_2_ adsorbents. Additional research is needed to improve their net advantages under more practical operating conditions and ultimately reduce reliance on costly structural tank material and high pressurization. This study also underscores the importance of investigating the stoichiometry of feedstock reactive groups in CMP design and synthesis, a variable currently lacking detailed investigation in existing literature. While only BH coupling was employed in this study, this approach should be explored with other chemistries to enable continued improvements in CMP porosity. PTPA networks have demonstrated practical utility in advancing H_2_ storage technologies, and future research should explore applying this optimization strategy to other cross‐coupling chemistries.

This study also underscores the importance of investigating the stoichiometry of feedstock reactive groups in CMP design and synthesis, a variable currently lacking detailed investigation in existing literature. While only BH coupling was employed in this study, this approach should be explored with other chemistries to enable continued improvements in CMP porosity. PTPA networks have demonstrated practical utility in advancing H_2_ storage technologies, and future research should explore applying this optimization strategy to other cross‐coupling chemistries.

## Conflict of Interest

The authors declare no conflict of interest.

## Supporting information



Supporting Information

Supporting Information

Supporting Information

Supporting Information

Supporting Information

Supporting Information

Supporting Information

Supporting Information

Supporting Information

Supporting Information

## Data Availability

The data that support the findings of this study are available in the supplementary material of this article.

## References

[1] H. Ishaq , I. Dincer , C. Crawford , Int. J. Hydrogen Energy 2022, 47, 26238.

[2] International Energy Agency, The Future of Hydrogen, https://www.iea.org/reports/the‐future‐of‐hydrogen (accessed: July 2024).

[3] L. Schlapbach , A. Züttel , Nature 2001, 414, 353. 11713542 10.1038/35104634

[4] R. Moradi , K. M. Groth , Int. J. Hydrogen Energy 2019, 44, 12254.

[5] M. Zhang , H. Lv , H. Kang , W. Zhou , C. Zhang , Int. J. Hydrogen Energy 2019, 44, 25777.

[6] E. Rivard , M. Trudeau , K. Zaghib , Materials 2019, 12, 1973. 31248099 10.3390/ma12121973 PMC6630991

[7] S. S. Samantaray , S. T. Putnam , N. P. Stadie , Inorganics 2021, 9, 45.

[8] U.S. Department of Energy, Target Explanation Document: Onboard Hydrogen Storage for Light‐Duty Fuel Cell Vehicles, https://www.energy.gov/sites/prod/files/2017/05/f34/fcto_targets_onboard_hydro_storage_explanation.pdf (accessed: July 2024).

[9] Toyota, Toyota Mirai technical specifications vs FCHV‐adv, https://mag.toyota.co.uk/toyota‐mirai‐technical‐specifications‐vs‐fchv‐adv/ (accessed: July 2024).

[10] F. Ding , B. I. Yakobson , Front. Phys. 2011, 6, 142.

[11] V. P. Ting , A. J. Ramirez‐Cuesta , N. Bimbo , J. E. Sharpe , A. Noguera‐Diaz , V. Presser , S. Rudic , T. J. Mays , ACS Nano 2015, 9, 8249. 26171656 10.1021/acsnano.5b02623

[12] L. R. Terry , S. Rols , M. Tian , I. da Silva , S. J. Bending , V. P. Ting , Nanoscale 2022, 14, 7250. 35521741 10.1039/d2nr00587e

[13] P. Bénard , R. Chahine , Scr. Mater. 2007, 56, 803.

[14] K. S. W. Sing , Pure Appl. Chem. 1985, 57, 603.

[15] M. Thommes , K. Kaneko , A. V. Neimark , J. P. Olivier , F. Rodriguez‐Reinoso , J. Rouquerol , K. S. W. Sing , Pure Appl. Chem. 2015, 87, 1051.

[16] S. K. Bhatia , A. L. Myers , Langmuir 2006, 22, 1688. 16460092 10.1021/la0523816

[17] M. Rzepka , P. Lamp , M. A. de la Casa‐Lillo , J. Phys. Chem. B 1998, 102, 10894.

[18] K. Kadono , H. Kajiura , M. Shiraishi , Appl. Phys. Lett. 2003, 83, 3392.

[19] S. Bai , M. Piri , Int. J. Hydrogen Energy 2022, 47, 24886.

[20] A. Thomas , Nat. Commun. 2020, 11, 4985. 33009410 10.1038/s41467-020-18746-5 PMC7532163

[21] D. P. Broom , C. J. Webb , K. E. Hurst , P. A. Parilla , T. Gennett , C. M. Brown , R. Zacharia , E. Tylianakis , E. Klontzas , G. E. Froudakis , T. h. A. Steriotis , P. N. Trikalitis , D. L. Anton , B. Hardy , D. Tamburello , C. Corgnale , B. A. van Hassel , D. Cossement , R. Chahine , M. Hirscher , Appl. Phys. A 2016, 122, 151.

[22] C. J. Rhodes , Annu. Rep. Prog. Chem., Sect. C: Phys. Chem. 2007, 103, 287.

[23] H. W. Langmi , D. Book , A. Walton , S. R. Johnson , M. M. Al‐Mamouri , J. D. Speight , P. P. Edwards , I. R. Harris , P. A. Anderson , J. Alloys Compd. 2005, 404–406, 637.

[24] M. Tian , M. J. Lennox , A. J. O'Malley , A. J. Porter , B. Krüner , S. Rudić , T. J. Mays , T. Düren , V. Presser , L. R. Terry , S. Rols , Y. Fang , Z. Dong , S. Rochat , V. P. Ting , Carbon 2021, 173, 968.

[25] J. L. Rowlandson , K. J. Edler , M. Tian , V. P. Ting , ACS Sustainable Chem. Eng. 2020, 8, 2186.

[26] S. Stock , N. Kostoglou , J. Selinger , S. Spirk , C. Tampaxis , G. Charalambopoulou , T. Steriotis , C. Rebholz , C. Mitterer , O. Paris , ACS Appl. Energy Mater. 2022, 5, 10915.

[27] C. D. Brewster , L. R. Terry , H. V. Doan , S. Rochat , V. P. Ting , Energy Adv. 2023, 2, 398.

[28] K. Geng , T. He , R. Liu , S. Dalapati , K. T. Tan , Z. Li , S. Tao , Y. Gong , Q. Jiang , D. Jiang , Chem. Rev. 2020, 120, 8814. 31967791 10.1021/acs.chemrev.9b00550

[29] D. Zhao , X. Wang , L. Yue , Y. He , B. Chen , Chem. Commun. 2022, 58, 11059. 10.1039/d2cc04036k 36112013

[30] S. P. Shet , S. Shanmuga Priya , K. Sudhakar , M. Tahir , Int. J. Hydrogen Energy 2021, 46, 11782.

[31] H. L. B. Boström , S. Emmerling , F. Heck , C. Koschnick , A. J. Jones , M. J. Cliffe , R. Al Natour , M. Bonneau , V. Guillerm , O. Shekhah , M. Eddaoudi , J. Lopez‐Cabrelles , S. Furukawa , M. Romero‐Angel , C. Martí‐Gastaldo , M. Yan , A. J. Morris , I. Romero‐Muñiz , Y. Xiong , A. E. Platero‐Prats , J. Roth , W. L. Queen , K. S. Mertin , D. E. Schier , N. R. Champness , H. H.‐M. Yeung , B. V. Lotsch , Adv. Mater. 2024, 36, 2304832. 10.1002/adma.202304832 37669645

[32] J.‐S. M. Lee , A. I. Cooper , Chem. Rev. 2020, 120, 2171. 31990527 10.1021/acs.chemrev.9b00399 PMC7145355

[33] A. I. Cooper , Adv. Mater. 2009, 21, 1291.

[34] Y. Liao , J. Weber , C. F. J. Faul , Chem. Commun. 2014, 50, 8002. 10.1039/c4cc03026e 24915169

[35] J.‐X. Jiang , F. Su , A. Trewin , C. D. Wood , N. L. Campbell , H. Niu , C. Dickinson , A. Y. Ganin , M. J. Rosseinsky , Y. Z. Khimyak , A. I. Cooper , Angew. Chem., Int. Ed. 2007, 46, 8574. 10.1002/anie.200701595 17899616

[36] Q. Chen , M. Luo , P. Hammershøj , D. Zhou , Y. Han , B. W. Laursen , C.‐G. Yan , B.‐H. Han , J. Am. Chem. Soc. 2012, 134, 6084. 22455734 10.1021/ja300438w

[37] J. L. C. Rowsell , A. R. Millward , K. S. Park , O. M. Yaghi , J. Am. Chem. Soc. 2004, 126, 5666. 15125649 10.1021/ja049408c

[38] Z. Wang , L. Sun , F. Xu , H. Zhou , X. Peng , D. Sun , J. Wang , Y. Du , Int. J. Hydrogen Energy 2016, 41, 8489.

[39] W. Huang , C. Gu , T. Wang , C. Gu , S. Qiao , R. Yang , RSC Adv. 2014, 4, 62525.

[40] Y. Xu , Z. Li , F. Zhang , X. Zhuang , Z. Zeng , J. Wei , RSC Adv. 2016, 6, 30048.

[41] C. Gu , Y. Bao , W. Huang , D. Liu , R. Yang , Macromol. Chem. Phys. 2016, 217, 748.

[42] M. Trunk , A. Herrmann , H. Bildirir , A. Yassin , J. Schmidt , A. Thomas , Chem. – Eur. J. 2016, 22, 7179. 27080951 10.1002/chem.201600783

[43] C.‐J. Sun , X.‐Q. Zhao , P.‐F. Wang , H. Wang , B.‐H. Han , Sci. China: Chem. 2017, 60, 1067.

[44] Y. Liao , Z. Cheng , M. Trunk , A. Thomas , Polym. Chem. 2017, 8, 7240.

[45] K. V. Rao , S. Mohapatra , C. Kulkarni , T. K. Maji , S. J. George , J. Mater. Chem. 2011, 21, 12958.

[46] S. Yuan , B. Dorney , D. White , S. Kirklin , P. Zapol , L. Yu , D.‐J. Liu , Chem. Commun. 2010, 46, 4547. 10.1039/c0cc00235f 20502839

[47] J. Schmidt , M. Werner , A. Thomas , Macromolecules 2009, 42, 4426.

[48] J.‐X. Jiang , A. Trewin , D. J. Adams , A. I. Cooper , Chem. Sci. 2011, 2, 1777.

[49] A. Suzuki , Chem. Commun. 2005, 4759. 10.1039/b507375h 16193109

[50] L. Chen , Y. Honsho , S. Seki , D. Jiang , J. Am. Chem. Soc. 2010, 132, 6742. 20218681 10.1021/ja100327h

[51] A. S. Guram , R. A. Rennels , S. L. Buchwald , Angew. Chem., Int. Ed. Engl. 1995, 34, 1348.

[52] J. Louie , J. F. Hartwig , Tetrahedron Lett. 1995, 36, 3609.

[53] M. Beygisangchin , S. Abdul Rashid , S. Shafie , A. R. Sadrolhosseini , H. N. Lim , Polymers 2021, 13, 2003. 34207392 10.3390/polym13122003 PMC8234317

[54] J. Chen , W. Yan , E. J. Townsend , J. Feng , L. Pan , V. Del Angel Hernandez , C. F. J. Faul , Angew. Chem., Int. Ed. 2019, 58, 11715. 10.1002/anie.201905488 PMC6771584 31206908

[55] J.‐X. Jiang , F. Su , A. Trewin , C. D. Wood , H. Niu , J. T. A. Jones , Y. Z. Khimyak , A. I. Cooper , J. Am. Chem. Soc. 2008, 130, 7710. 18500800 10.1021/ja8010176

[56] L. Pan , Z. Liu , M. Tian , B. C. Schroeder , A. E. Aliev , C. F. J. Faul , ACS Appl. Mater. Interfaces 2019, 11, 48352. 31789014 10.1021/acsami.9b16767

[57] J. Chen , T. Qiu , W. Yan , C. F. J. Faul , J. Mater. Chem. A 2020, 8, 22657.

[58] C. M. Hansen , Eur. Polym. J. 2008, 44, 2741.

[59] P. Kubelka , F. Munk , Z. Phys. 1931, 12, 259.

[60] A. K. Roy Choudhury , In Principles of Colour and Appearance Measurement, (Ed: A. K. Roy Choudhury ), Woodhead Publishing, Oxford, UK 2015, 117.

[61] International Organization for Standardization, ISO 11358‐1:2022 Plastics — Thermogravimetry (TG) of polymers — Part 1: General principles, https://www.iso.org/standard/79999.html (accessed: July 2024).

[62] S. Brunauer , P. H. Emmett , E. Teller , J. Am. Chem. Soc. 1938, 60, 309.

[63] J. Rouquerol , P. Llewellyn , F. Rouquerol , In Studies in Surface Science and Catalysis, (Eds: P. L. Llewellyn , F. Rodriquez‐Reinoso , J. Rouqerol , N. Seaton ), Elsevier, New York 2007, 49.

[64] International Organization for Standardization, ISO 9277:2022 Determination of the specific surface area of solids by gas adsorption — BET method, https://www.iso.org/standard/71014.html (accessed: July 2024).

[65] J. P. Olivier , J. Porous Mater. 1995, 2, 9.

[66] F. Rouquerol , J. Rouquerol , K. S. W. Sing , P. L. Llewellyn , G. Maurin , Adsorption by Powders and Porous Solids: Principles, Methodology and Applications, 2nd ed., Elsevier/AP, Amsterdam, NL 2014.

[67] A. L. Myers , P. A. Monson , Adsorption 2014, 20, 591.

[68] D. P. Broom , Adsorption 2024, 30, 1565.

[69] J. A. Mason , M. Veenstra , J. R. Long , Chem. Sci. 2013, 5, 32.

[70] D. P. Broom , K. M. Thomas , MRS Bull. 2013, 38, 412.

[71] E. W. Lemmon , I. H. Bell , M. L. Huber , M. O. McLinden , NIST Standard Reference Database Vol. 23: Reference Fluid Thermodynamic and Transport Properties‐REFPROP, Version 9.1, National Institute of Standards and Technology, https://www.nist.gov/srd/refprop (accessed: July 2024).

[72] J. W. Leachman , R. T. Jacobsen , S. G. Penoncello , E. W. Lemmon , J. Phys. Chem. Ref. Data 2009, 38, 721.

[73] S. Gumma , O. Talu , Langmuir 2010, 26, 17013. 20886890 10.1021/la102186q

[74] E. Glueckauf , Trans. Faraday Soc. 1955, 51, 1540.

[75] D. L. Pavia , G. M. Lampman , G. S. Kriz , J. R. Vyvyan , Introduction to Spectroscopy, 5th ed., Cengage Learning, Stamford, CT 2015.

[76] X. Ding , B.‐H. Han , Angew. Chem., Int. Ed. 2015, 54, 6536. 10.1002/anie.201501732 25873264

[77] A. Gibaud , J. S. Xue , J. R. Dahn , Carbon 1996, 34, 499.

[78] A. Guinier , G. Fournet , Small‐angle scattering of X‐rays, Wiley, New York, USA, 1955.

[79] C. J. Gommes , T. Asset , J. Drnec , J. Appl. Crystallogr. 2019, 52, 507.

[80] H. Schnablegger , Y. Singh , The SAXS Guide: Getting acquainted with the principles, 5th ed., Anton Paar GmbH, Graz, Austria, 2023.

[81] J. E. Martin , A. J. Hurd , J. Appl. Crystallogr. 1987, 20, 61.

[82] H. R. Schubach , E. Nagy , B. Heise , Colloid Polym. Sci. 1981, 259, 789.

[83] V. Velev , A. Popov , P. Kyurkchiev , L. Veleva , M. Pencheva , J. Phys.: Conf. Ser. 2014, 558, 012051.

[84] G. Mason , J. Colloid Interface Sci. 1982, 88, 36. 10.1006/jcis.1996.0009 10479420

[85] M. Parlar , Y. C. Yortsos , J. Colloid Interface Sci. 1989, 132, 425.

[86] G. C. Wall , R. J. C. Brown , J. Colloid Interface Sci. 1981, 82, 141.

[87] F. Rojas , I. Kornhauser , C. Felipe , J. M. Esparza , S. Cordero , A. Domínguez , J. L. Riccardo , Phys. Chem. Chem. Phys. 2002, 4, 2346.

[88] J. D. Evans , V. Bon , I. Senkovska , S. Kaskel , Langmuir 2021, 37, 4222. 33797923 10.1021/acs.langmuir.1c00122

[89] R. M. Kassab , K. T. Jackson , O. M. El‐Kadri , H. M. El‐Kaderi , Res. Chem. Intermed. 2011, 37, 747.

[90] T. E. Reich , K. T. Jackson , S. Li , P. Jena , H. M. El‐Kaderi , J. Mater. Chem. 2011, 21, 10629.

[91] M. I. Maulana Kusdhany , S. M. Lyth , Carbon 2021, 179, 190.

[92] J. E. Sharpe , N. Bimbo , V. P. Ting , A. D. Burrows , D. Jiang , T. J. Mays , Adsorption 2013, 19, 643.

[93] Y. Gogotsi , C. Portet , S. Osswald , J. M. Simmons , T. Yildirim , G. Laudisio , J. E. Fischer , Int. J. Hydrogen Energy 2009, 34, 6314.

[94] N. Bimbo , W. Xu , J. E. Sharpe , V. P. Ting , T. J. Mays , Mater. Des. 2016, 89, 1086.

[95] G. E. Decker , E. D. Bloch , ACS Appl. Mater. Interfaces 2021, 13, 51925. 34156822 10.1021/acsami.1c07304

[96] M. E. Ergün , S. Bulbul , Int. Adv. Res. Eng. J. 2022, 6, 167.

[97] M. Tian , S. Rochat , K. Polak‐Kraśna , L. T. Holyfield , A. D. Burrows , C. R. Bowen , T. J. Mays , Adsorption 2019, 25, 889.

[98] N. B. McKeown , P. M. Budd , Chem. Soc. Rev. 2006, 35, 675. 16862268 10.1039/b600349d

[99] A. Bhunia , V. Vasylyeva , C. Janiak , Chem. Commun. 2013, 49, 3961. 10.1039/c3cc41382a 23563918

[100] S. Qiao , W. Huang , Z. Du , X. Chen , F.‐K. Shieh , R. Yang , New J. Chem. 2014, 39, 136.

[101] W. Zhang , C. Li , Y.‐P. Yuan , L.‐G. Qiu , A.‐J. Xie , Y.‐H. Shen , J.‐F. Zhu , J. Mater. Chem. 2010, 20, 6413.

[102] Q. Chen , D.‐P. Liu , M. Luo , L.‐J. Feng , Y.‐C. Zhao , B.‐H. Han , Small 2014, 10, 308. 23913850 10.1002/smll.201301618

[103] J. L. C. Rowsell , A. R. Millward , K. S. Park , O. M. Yaghi , J. Am. Chem. Soc. 2004, 126, 5666. 15125649 10.1021/ja049408c

[104] O. K. Farha , A. Özgür Yazaydın , I. Eryazici , C. D. Malliakas , B. G. Hauser , M. G. Kanatzidis , S. T. Nguyen , R. Q. Snurr , J. T. Hupp , Nat. Chem. 2010, 2, 944. 20966950 10.1038/nchem.834

[105] M. Hirscher , L. Zhang , H. Oh , Appl. Phys. A: Mater. Sci. Process. 2023, 129, 112.

[106] A. Hruzewicz‐Kołodziejczyk , V. P. Ting , N. Bimbo , T. J. Mays , Int. J. Hydrogen Energy 2012, 37, 2728. 10.1039/c0fd00010h 22455063

[107] D. P. Broom , C. J. Webb , K. E. Hurst , P. A. Parilla , T. Gennett , C. M. Brown , R. Zacharia , E. Tylianakis , E. Klontzas , G. E. Froudakis , T. h. A. Steriotis , P. N. Trikalitis , D. L. Anton , B. Hardy , D. Tamburello , C. Corgnale , B. A. van Hassel , D. Cossement , R. Chahine , M. Hirscher , Appl. Phys. A: Mater. Sci. Process. 2016, 122, 151.

