# Supplementary material for: Polytriphenylamine Conjugated Microporous Polymers as Versatile Platforms for Tunable Hydrogen Storage
Source: Small. 2024 Oct 31;21(13):2407292. doi: 10.1002/smll.202407292 (PMC11962686; doi:10.1002/smll.202407292)
Supplement: Supplementary file 1 — Supporting Information [file SMLL-21-2407292-s006.pdf]

NANO · MICRO
small

Supporting Information

for *Small*, DOI 10.1002/smll.202407292

Polytriphenylamine Conjugated Microporous Polymers as Versatile Platforms for Tunable Hydrogen Storage

*John D. Worth, Annela M. Seddon, Valeska P. Ting and Charl F. J. Faul**

Polytriphenylamine Conjugated Microporous Polymers as Versatile Platforms for Tunable Hydrogen Storage

John D. Worth, Annela M. Seddon, Valeska P. Ting, and Charl F. J. Faul

Contents

1 Reagent characterization **2**
1.1 Nuclear magnetic resonance spectroscopy . . . 2
1.1.1 Proton nuclear magnetic resonance spectroscopy . . . 2
1.1.2 Carbon-13 nuclear magnetic resonance spectroscopy . . . 4
1.1.3 Fluorine-19 nuclear magnetic resonance spectroscopy . . . 7
1.2 Fourier-transform infrared spectroscopy . . . 7

2 Powder X-ray diffraction sample holder diffraction pattern **10**

3 Excess and total adsorption **10**

4 Total adsorption assumptions **10**

5 Isothermal properties of hydrogen **12**

6 Synthesis of conjugated microporous polytriphenylamines **12**
6.1 Sample naming convention . . . 12
6.2 PTPA-Br_1N_1 . . . 13
6.3 PTPA-$Br_{1.5}N_1$ . . . 13
6.4 PTPA-$Br_{2.25}N_1$ . . . 13
6.5 PTPA-Br_3N_1 . . . 13
6.6 PTPA-$Br_1N_{1.5}$ . . . 13
6.7 PTPA-$Br_1N_{2.25}$ . . . 13
6.8 PTPA-Br_1N_3 . . . 14

7 Photograph of PTPA **14**

8 Diffuse reflectance of PTPAs **15**

9 Thermal degradation of PTPAs **15**

10 Fractal dimension of PTPAs **16**

11 Nitrogen adsorption isotherms presented with a logarithm scale **17**

12 Determination of specific surface areas **17**

13 Hydrogen sorption isotherms **19**
13.1 Isotherms determined volumetrically at low-pressures . . . 19
13.2 Isotherms determined gravimetrically at high-pressures . . . 22
13.3 High-pressure total isotherms . . . 25
13.4 High-pressure net isotherms . . . 28

14 Helium pycnometry **31**

15 Fractional transient uptake plots, linear driving force fittings and calculated rates **32**

1 Reagent characterization

An expanded characterization of the reagents used in the Buchwald–Hartwig amination is provided here.

1.1 Nuclear magnetic resonance spectroscopy

Nuclear magnetic resonance (NMR) spectra were recorded on a JEOL ECZ400 Fourier transform NMR spectrometer fitted with a Royal HFX probe and operating at frequency 400 MHz. Approximately 10–15 mg of sample was dissolved in deuterated chloroform ($CDCl_3$) or deuterated water (D_2O) and transferred to Norell Standard Series 5 mm NMR tubes (part number: 502-7).

The chemical shifts (δ) are reported in parts per million (ppm) relative to the residual solvent peak e.g., 7.26 ppm for $CDCl_3$ and 4.79 ppm for D_2O.

^{1}H-NMR spectra were acquired with the following parameters: a pulse width of 2.21 µs, relaxation delay of 2 s, and acquisition time of 2 s. A total of 8 scans were accumulated to ensure adequate signal-to-noise ratio.

^{13}C-NMR spectra were acquired with the following parameters: a pulse width of 3.71 µs, relaxation delay of 2 s, and acquisition time of 1 s. A total of 1024 scans were accumulated to ensure adequate signal-to-noise ratio. Proton decoupling was applied.

^{19}F-NMR spectra were acquired with the following parameters: a pulse width of 3.71 µs, relaxation delay of 2 s, and acquisition time of 2 s. A total of 16 scans were accumulated to ensure adequate signal-to-noise ratio.

All measurements were performed at approximately 22 °C.

1.1.1 Proton nuclear magnetic resonance spectroscopy

Reagents involved in the synthesis of PTPA as characterized by ^{1}H-NMR.

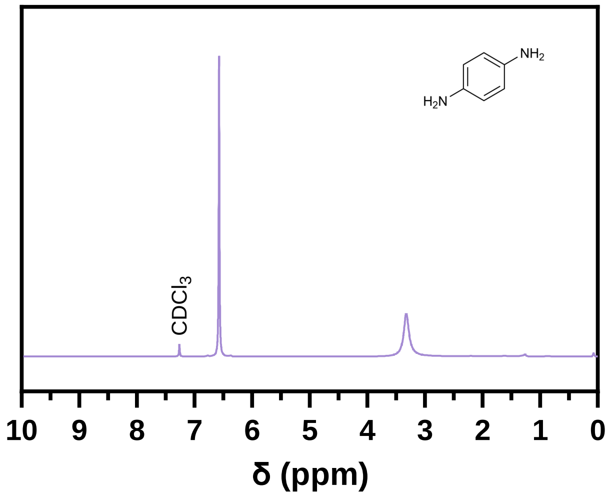

Figure S1: ^{1}H-NMR (400 MHz, $CDCl_3$) spectrum of *p*-phenylenediamine. Solvent residual peak is labelled. Molecule structure is also shown.

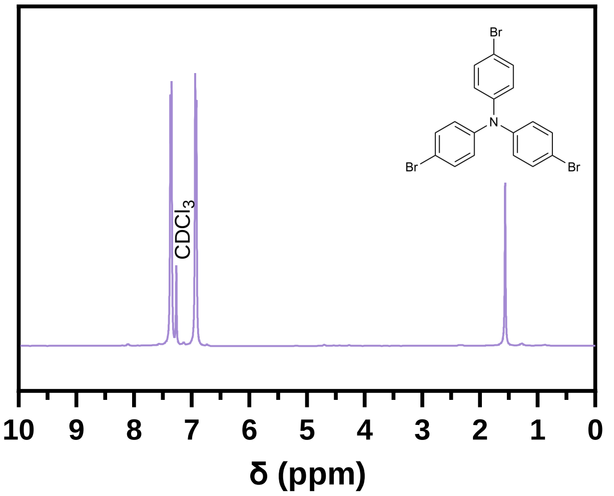

Figure S2: ^{1}H-NMR (400 MHz, $CDCl_3$) spectrum of tris(4-bromophenyl)amine. Solvent residual peak is labelled. Molecule structure is also shown.

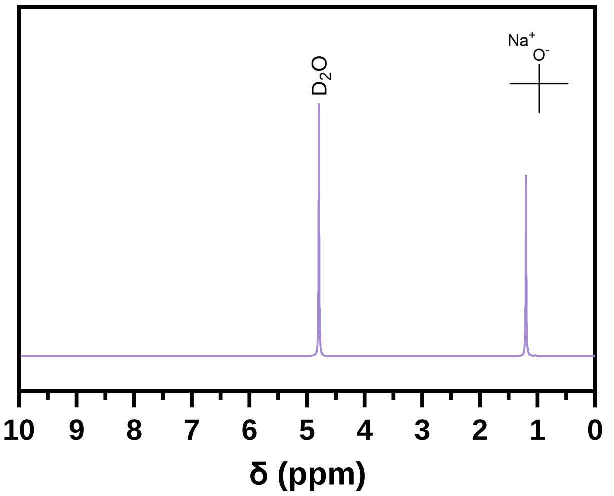

Figure S3: ^{1}H-NMR (400 MHz, D_2O) spectrum of sodium *tert*-butoxide. Solvent residual peak is labelled. Molecule structure is also shown.

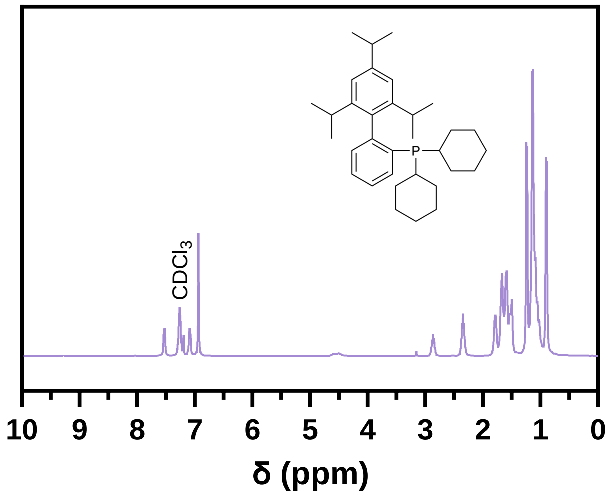

Figure S4: ^{1}H-NMR (400 MHz, $CDCl_3$) spectrum of dicyclohexyl[2′,4′,6′-tris(propan-2-yl)[1,1′-biphenyl]-2-yl]phosphane (XPhos). Solvent residual peak is labelled. Molecule structure is also shown.

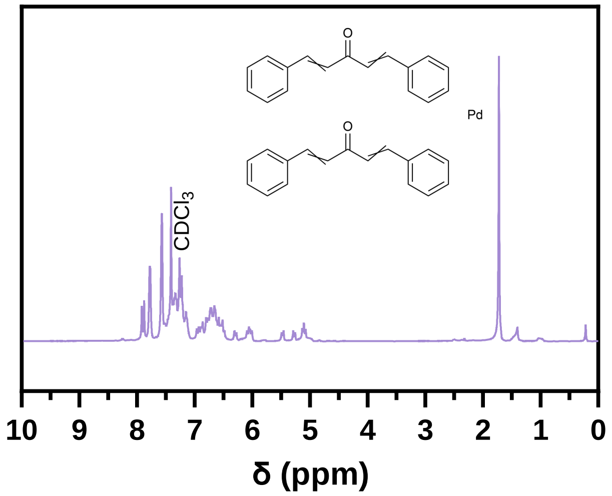

Figure S5: ^{1}H-NMR (400 MHz, $CDCl_3$) spectrum of bis(dibenzylideneacetone)palladium(0)-($Pd(dba)_2$). Solvent residual peak is labelled. Molecule structure is also shown.

1.1.2 Carbon-13 nuclear magnetic resonance spectroscopy

Reagents involved in the synthesis of PTPA as characterized by ^{13}C-NMR.

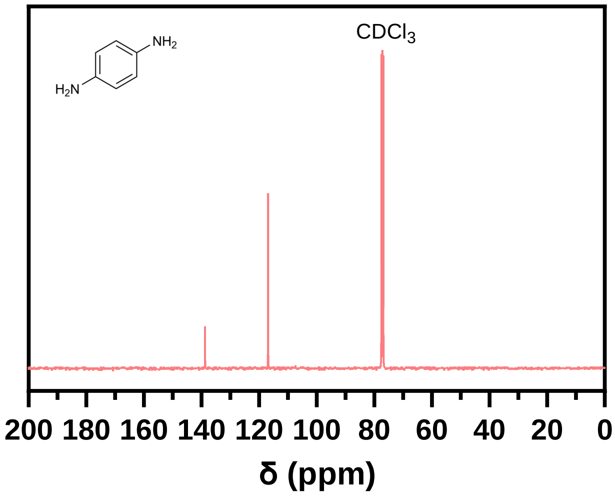

Figure S6: ^{13}C-NMR (400 MHz, $CDCl_3$) spectrum of *p*-phenylenediamine. Solvent residual peak is labelled. Molecule structure is also shown.

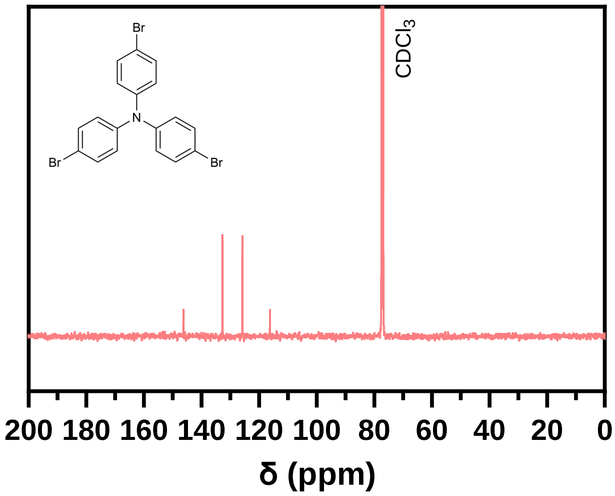

Figure S7: ^{13}C-NMR (400 MHz, $CDCl_3$) spectrum of tris(4-bromophenyl)amine. Solvent residual peak is labelled. Molecule structure is also shown.

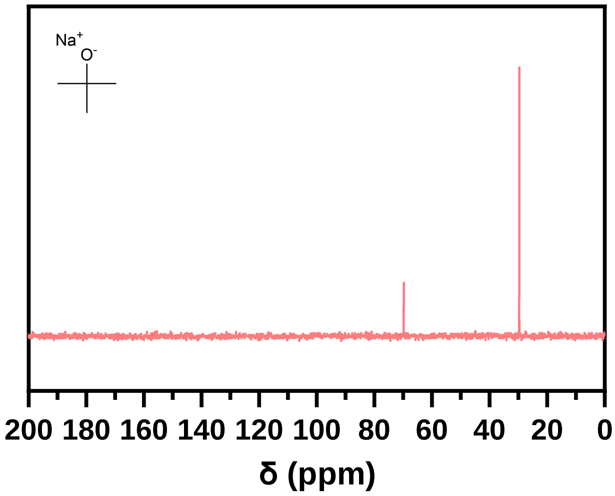

Figure S8: ^{13}C-NMR (400 MHz, D_2O) spectrum of sodium *tert*-butoxide. Molecule structure is also shown.

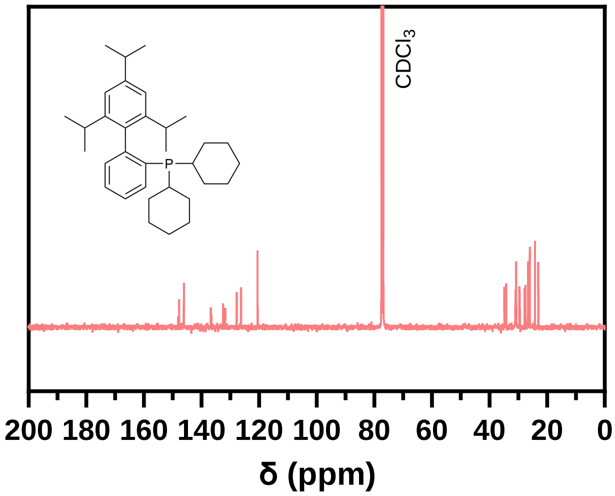

Figure S9: ^{13}C-NMR (400 MHz, $CDCl_3$) spectrum of dicyclohexyl[2′,4′,6′-tris(propan-2-yl)[1,1′-biphenyl]-2-yl]phosphane (XPhos). Solvent residual peak is labelled. Molecule structure is also shown.

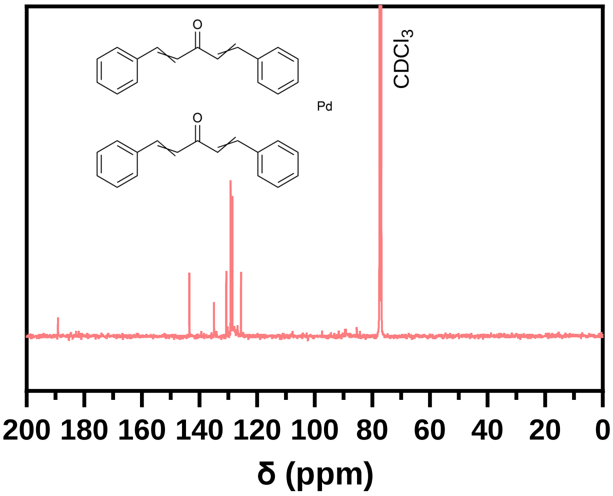

Figure S10: ^{13}C-NMR (400 MHz, $CDCl_3$) spectrum of bis(dibenzylideneacetone)palladium(0)-($Pd(dba)_2$). Solvent residual peak is labelled. Molecule structure is also shown.

1.1.3 Fluorine-19 nuclear magnetic resonance spectroscopy

Reagents involved in the synthesis of PTPA as characterized by ^{19}F-NMR.

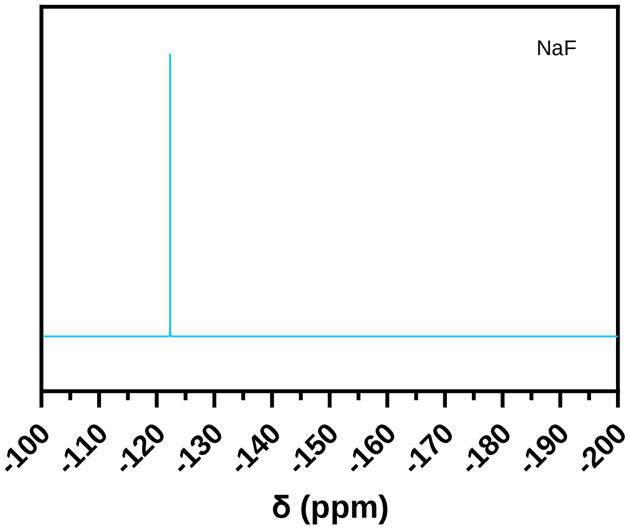

Figure S11: ^{19}F-NMR (400 MHz, D_2O) spectrum of sodium fluoride (NaF). Molecule structure is also shown.

1.2 Fourier-transform infrared spectroscopy

All other reagents involved in PTPA synthesis and not shown in the main manuscript as characterized by FTIR. The spectra below were recorded using a PerkinElmer FTIR Spectrometer Spectrum Two equipped with a lithium tantalate ($LiTaO_3$) detector and a diamond crystal in attenuated total reflection (ATR) geometry. An accumulation of 25 scans was performed in the wavenumber range of 4000–450 cm^{-1} at a resolution of 4 cm^{-1}. The background spectrum was measured separately and then subtracted. A baseline correction was performed on all collected spectra.

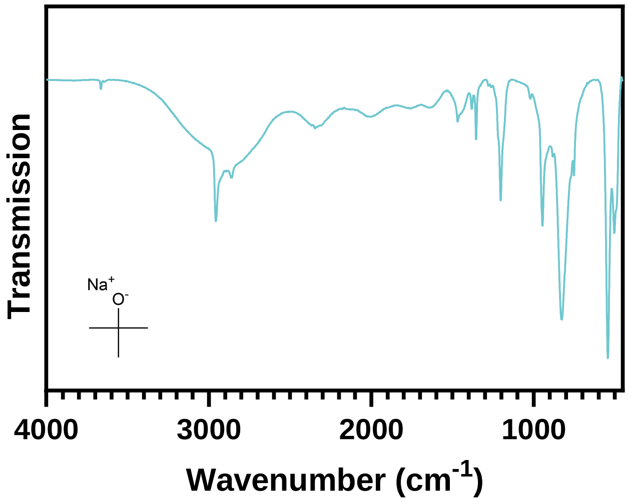

Figure S12: FTIR spectrum of sodium *tert*-butoxide (NaOtBu) between wavenumbers 4000–450 $\mathrm{cm^{-1}}$. Molecule structure is also shown.

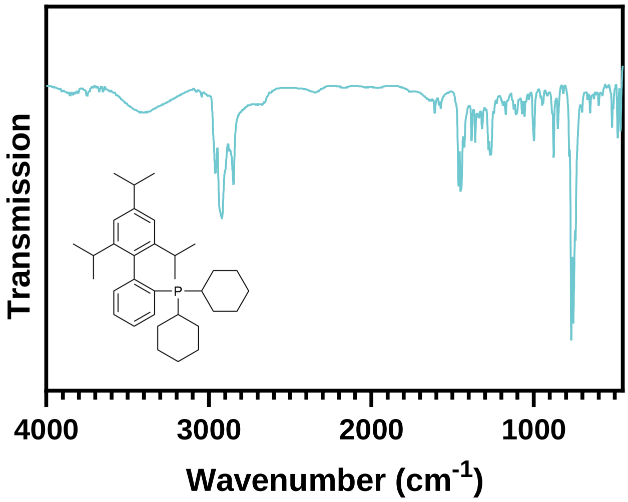

Figure S13: FTIR spectrum of dicyclohexyl[2′,4′,6′-tris(propan-2-yl)[1,1′-biphenyl]-2-yl]phosphane (XPhos) between wavenumbers 4000–450 $\mathrm{cm^{-1}}$. Molecule structure is also shown.

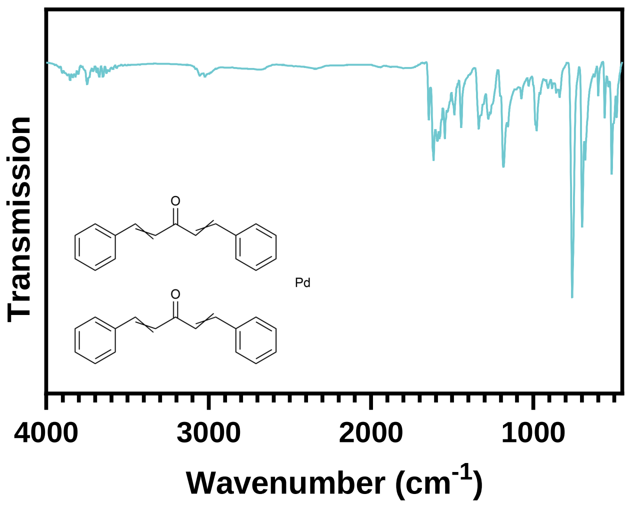

Figure S14: FTIR spectrum of bis(dibenzylideneacetone)palladium(0) ($Pd(dba)_2$) between wavenumbers 4000–450 cm^{-1}. Molecule structure is also shown.

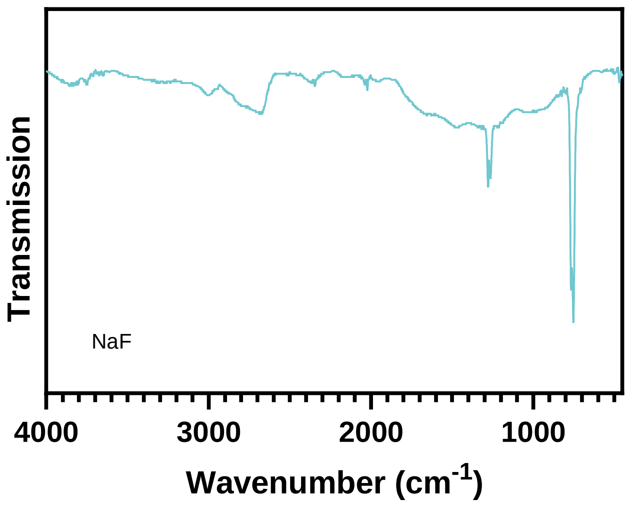

Figure S15: FTIR spectrum of sodium fluoride (NaF) between wavenumbers 4000–450 cm^{-1}. Molecule structure is also shown.

2 Powder X-ray diffraction sample holder diffraction pattern

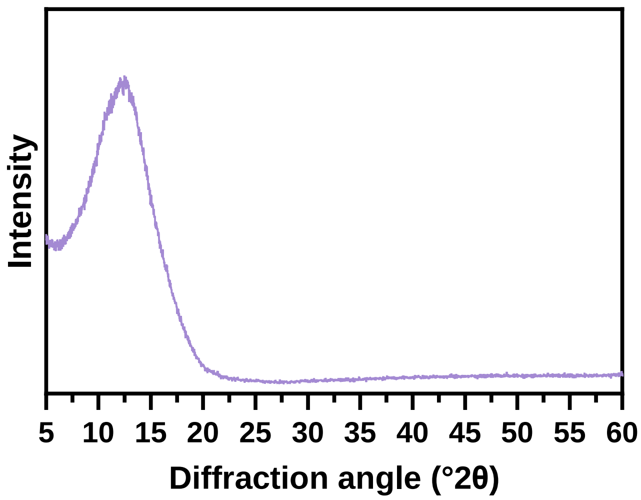

Figure S16: Diffraction pattern of the silicon powder sample holder utilized during polymer characterization.

3 Excess and total adsorption

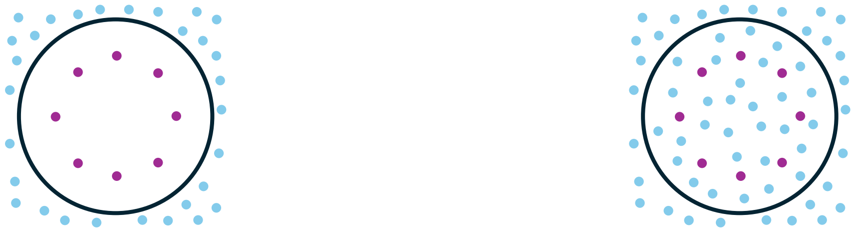

(a) Gibbs surface excess adsorption.

(b) Total adsorption.

Figure S17: A schematic illustration of the two reference states for (a) excess and (b) total adsorption on a Gibbs dividing surface using the context of a slit pore (represented by the black circle). The purple circles (•) represent the excess adsorbed H_2 on a CMP. The blue circles (•) represent the bulk, non-adsorbed H_2 molecules. Only H_2 inside the slit pore is considered in the total adsorption calculation.

4 Total adsorption assumptions

These figures are intended to help visualise and explain the two correction methods for absolute adsorption defined within the main manuscript. Equation 1 in the manuscript is known as the constant volume correction (Figure S18) and Equation 2 as the constant density correction (Figure S19).

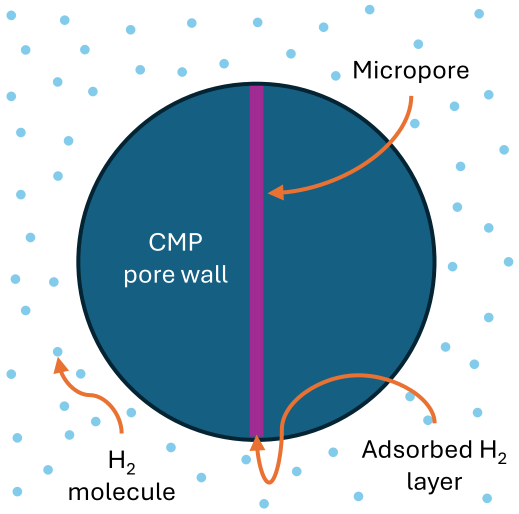

Figure S18: The constant volume correction presumes that all pore volume is excluded. This aligns with the constant density correction when pores reach saturation. The pore volume considered in this correction is, by definition, the micropore volume estimated or determined through alternative methods such as N_2 gas sorption analysis.

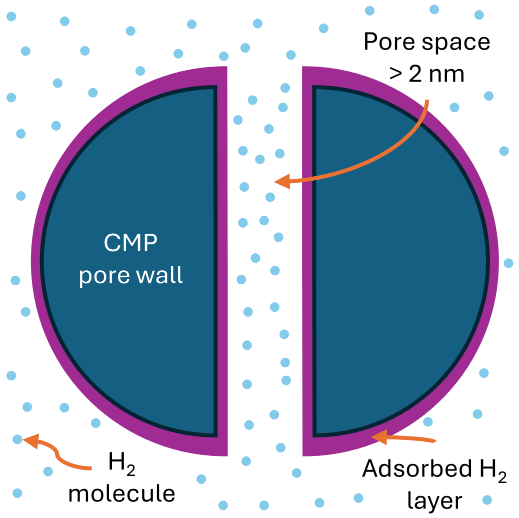

Figure S19: The absolute adsorption correction with constant density assumes that the H_2 adsorbate occupies pore spaces until they are filled and that the density of the adsorbed layer remains constant. The density of the adsorbed phase is frequently assumed to match that of the liquid phase of the adsorbate.

5 Isothermal properties of hydrogen

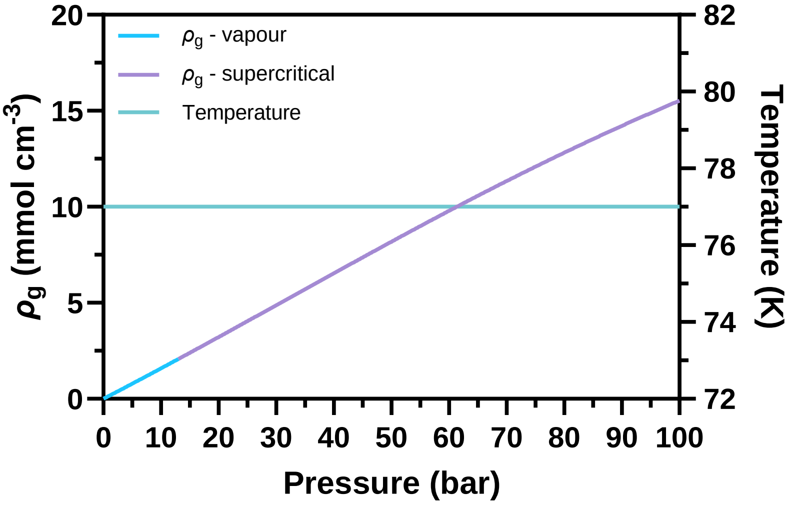

Figure S20: Density of H_2 in mmol cm^{-3} (ρ_g) versus pressures from 0–100 bar at 77 K. Different phases of H_2 are shown by the line colours: — for the vapour phase and — for the supercritical phase. Temperature is shown by the — line. Plot created using data from NIST. [1, 2]

6 Synthesis of conjugated microporous polytriphenylamines

Synthesis of all polytriphenylamine (PTPA) materials follows the general transformation via Buchwald—Hartwig amination in Scheme S1.

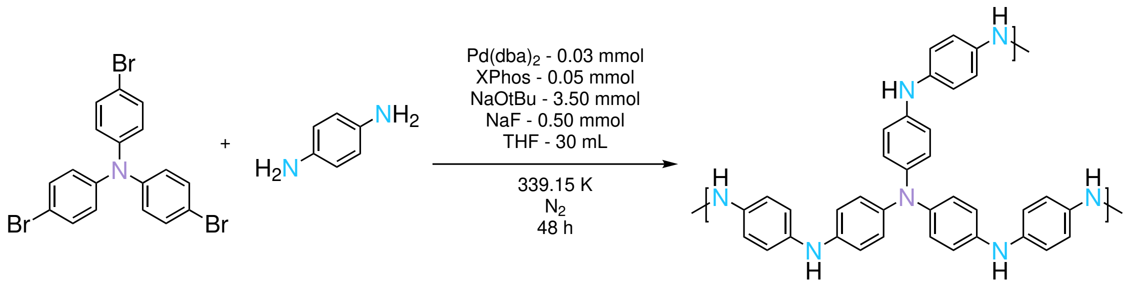

Scheme S1: General conditions used for the Buchwald—Hartwig amination cross-coupling reaction between monomers tris(4-bromophenyl)amine and *p*-phenylenediamine to form the idealized structure of PTPA.

6.1 Sample naming convention

Sample names were assigned to indicate the ratio of bromine atoms (Br_x) to amine functional groups (N_x) in the targeted PTPA CMP material, denoted as PTPA-Br_xN_x.

For instance, in a stoichiometric reaction where the number of reacting functional groups is equal, the amount of tris(4-bromophenyl)amine (C_3 monomer with 3 aryl halide sites) is balanced with the amount of *p*-phenylenediamine (C_2 monomer with 2 amine functional groups) to achieve an equal number of reactive species.

For example, 0.33 mmol of tris(4-bromophenyl)amine is multiplied by 1.5 to give 0.495 mmol of *p*-phenylenediamine (as $2 \times 1.5 = 3$), resulting in a $3:3$ ratio (simplified to $1:1$) of participating functional groups. This sample is named PTPA-Br_1N_1.

6.2 PTPA-Br_1N_1

A Schlenk tube was charged with tris(4-bromophenyl)amine (159.06 mg, 0.33 mmol), *p*-phenylenediamine (54.07 mg, 0.50 mmol), bis(dibenzylideneacetone)palladium(0) ($Pd(dba)_2$, 17.25 mg, 0.03 mmol), dicyclohexyl[2′,4′,6′-tris(propan-2-yl)[1,1′-biphenyl]-2-yl]phosphane (XPhos, 21.45 mg, 0.05 mmol), sodium *tert*-butoxide (NaOtBu, 336.35 mg, 3.50 mmol), and sodium fluoride (NaF, 20.99 mg, 0.50 mmol). Anhydrous tetrahydrofuran (THF, 30 mL) was added under a N_2 atmosphere. The reaction mixture was heated to 339 K with stirring and kept under an inert atmosphere for 48 h. The resulting product was collected by centrifugation before being purified with water (H_2O), methanol (CH_3OH), ethanol (C_2H_6O), and chloroform ($CHCl_3$, 300 mL each) to remove residual catalyst, impurities, and any oligomers. The insoluble polymeric materials were then dried under vacuum at 343 K for 24 h.

6.3 PTPA-$Br_{1.5}N_1$

A Schlenk tube was charged with tris(4-bromophenyl)amine (241.01 mg, 0.50 mmol), *p*-phenylenediamine (54.07 mg, 0.50 mmol), $Pd(dba)_2$ (17.25 mg, 0.03 mmol), XPhos (21.45 mg, 0.05 mmol), NaOtBu (336.35 mg, 3.50 mmol), and NaF (20.99 mg, 0.50 mmol). Anhydrous THF (30 mL) was added under a N_2 atmosphere. The reaction mixture was heated to 339 K with stirring and kept under an inert atmosphere for 48 h. The resulting product was collected by centrifugation before being purified with H_2O, CH_3OH, C_2H_6O, and $CHCl_3$ (300 mL each) to remove residual catalyst, impurities, and any oligomers. The insoluble polymeric materials were then dried under vacuum at 343 K for 24 h.

6.4 PTPA-$Br_{2.25}N_1$

A Schlenk tube was charged with tris(4-bromophenyl)amine (360.75 mg, 0.75 mmol), *p*-phenylenediamine (54.07 mg, 0.50 mmol), $Pd(dba)_2$ (17.25 mg, 0.03 mmol), XPhos (21.45 mg, 0.05 mmol), NaOtBu (336.35 mg, 3.50 mmol), and NaF (20.99 mg, 0.50 mmol). Anhydrous THF (30 mL) was added under a N_2 atmosphere. The reaction mixture was heated to 339 K with stirring and kept under an inert atmosphere for 48 h. The resulting product was collected by centrifugation before being purified with H_2O, CH_3OH, C_2H_6O, and $CHCl_3$ (300 mL each) to remove residual catalyst, impurities, and any oligomers. The insoluble polymeric materials were then dried under vacuum at 343 K for 24 h.

6.5 PTPA-Br_3N_1

A Schlenk tube was charged with tris(4-bromophenyl)amine (481.01 mg, 1.00 mmol), *p*-phenylenediamine (54.07 mg, 0.50 mmol), $Pd(dba)_2$ (17.25 mg, 0.03 mmol), XPhos (21.45 mg, 0.05 mmol), NaOtBu (336.35 mg, 3.50 mmol), and NaF (20.99 mg, 0.50 mmol). Anhydrous THF (30 mL) was added under a N_2 atmosphere. The reaction mixture was heated to 339 K with stirring and kept under an inert atmosphere for 48 h. The resulting product was collected by centrifugation before being purified with H_2O, CH_3OH, C_2H_6O, and $CHCl_3$ (300 mL each) to remove residual catalyst, impurities, and any oligomers. The insoluble polymeric materials were then dried under vacuum at 343 K for 24 h.

6.6 PTPA-$Br_1N_{1.5}$

A Schlenk tube was charged with tris(4-bromophenyl)amine (241.01 mg, 0.50 mmol), *p*-phenylenediamine (121.66 mg, 1.125 mmol), $Pd(dba)_2$ (17.25 mg, 0.03 mmol), XPhos (21.45 mg, 0.05 mmol), NaOtBu (336.35 mg, 3.50 mmol), and NaF (20.99 mg, 0.50 mmol). Anhydrous THF (30 mL) was added under a N_2 atmosphere. The reaction mixture was heated to 339 K with stirring and kept under an inert atmosphere for 48 h. The resulting product was collected by centrifugation before being purified with H_2O, CH_3OH, C_2H_6O, and $CHCl_3$ (300 mL each) to remove residual catalyst, impurities, and any oligomers. The insoluble polymeric materials were then dried under vacuum at 343 K for 24 h.

6.7 PTPA-$Br_1N_{2.25}$

A Schlenk tube was charged with tris(4-bromophenyl)amine (241.01 mg, 0.50 mmol), *p*-phenylenediamine (182.49 mg, 1.69 mmol), $Pd(dba)_2$ (17.25 mg, 0.03 mmol), XPhos (21.45 mg, 0.05 mmol), NaOtBu (336.35 mg, 3.50 mmol), and NaF (20.99 mg, 0.50 mmol). Anhydrous THF (30 mL) was

added under a N_2 atmosphere. The reaction mixture was heated to 339 K with stirring and kept under an inert atmosphere for 48 h. The resulting product was collected by centrifugation before being purified with H_2O, CH_3OH, C_2H_6O, and $CHCl_3$ (300 mL each) to remove residual catalyst, impurities, and any oligomers. The insoluble polymeric materials were then dried under vacuum at 343 K for 24 h.

6.8 PTPA-Br_1N_3

A Schlenk tube was charged with tris(4-bromophenyl)amine (241.01 mg, 0.50 mmol), *p*-phenylenediamine (243.3 mg, 2.25 mmol), $Pd(dba)_2$ (17.25 mg, 0.03 mmol), XPhos (21.45 mg, 0.05 mmol), NaOtBu (336.35 mg, 3.50 mmol), and NaF (20.99 mg, 0.50 mmol). Anhydrous THF (30 mL) was added under a N_2 atmosphere. The reaction mixture was heated to 339 K with stirring and kept under an inert atmosphere for 48 h. The resulting product was collected by centrifugation before being purified with H_2O, CH_3OH, C_2H_6O, and $CHCl_3$ (300 mL each) to remove residual catalyst, impurities, and any oligomers. The insoluble polymeric materials were then dried under vacuum at 343 K for 24 h.

7 Photograph of PTPA

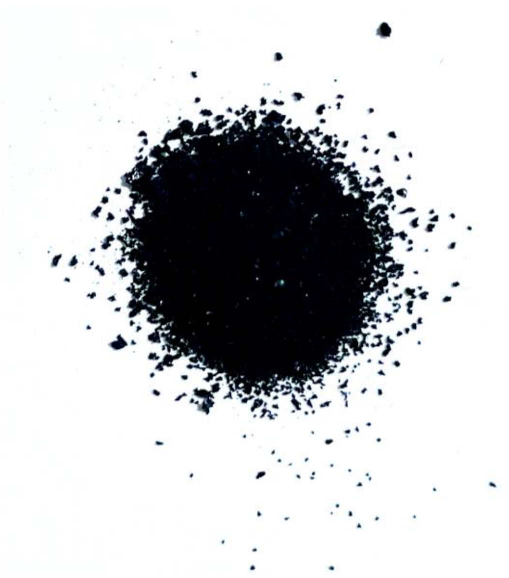

Figure S21: Digital photograph of PTPA-$Br_{2.25}N_1$, representative of the appearance of all PTPA samples.

8 Diffuse reflectance of PTPAs

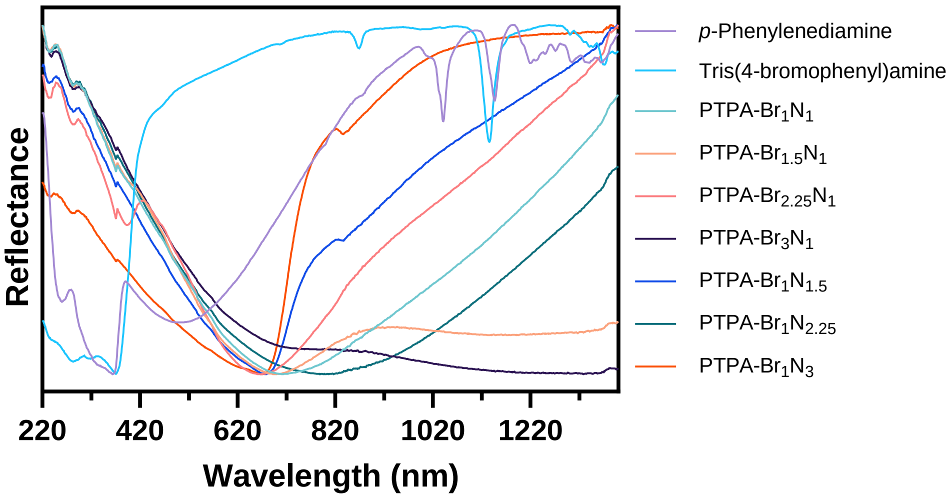

Figure S22: Measured diffuse reflectance of monomers and synthesized PTPAs.

9 Thermal degradation of PTPAs

Thermal onset decomposition temperatures (T_o) in N_2 were determined for each polymer using TGA and are summarized in Table S1.

Table S1: T_o values of PTPAs calculated from TGA studies.

CMP	T_o (°C)
PTPA-Br_1N_1	254
PTPA-$Br_{1.5}N_1$	448
PTPA-$Br_{2.25}N_1$	275
PTPA-Br_3N_1	398
PTPA-$Br_1N_{1.5}$	419
PTPA-$Br_1N_{2.25}$	380

10 Fractal dimension of PTPAs

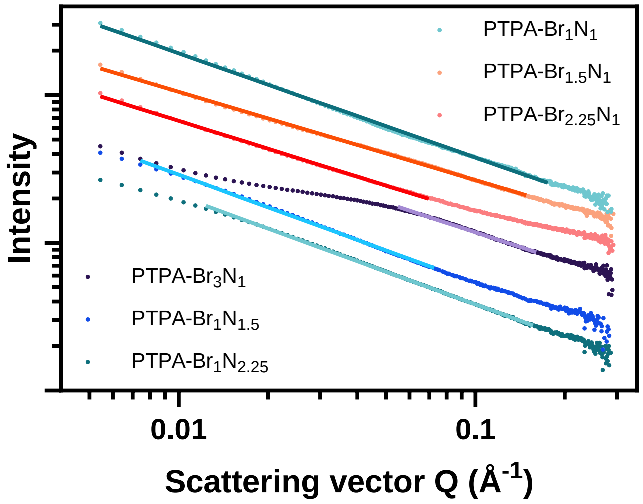

Figure S23: SAXS patterns of PTPAs were analyzed by fitting linear regressions (shown by the solid lines) to a selected linear region to determine the power law exponent (n).

Table S2: Summary of fractal dimension/power law exponent (n) for PTPAs, with adjusted R^2 values from the linear regressions.

CMP	n	Adjusted R^2
PTPA-Br_1N_1	3.52	0.99
PTPA-$Br_{1.5}N_1$	2.99	0.99
PTPA-$Br_{2.25}N_1$	3.14	0.99
PTPA-Br_3N_1	3.29	0.99
PTPA-$Br_1N_{1.5}$	3.67	0.99
PTPA-$Br_1N_{2.25}$	3.67	0.99

11 Nitrogen adsorption isotherms presented with a logarithm scale

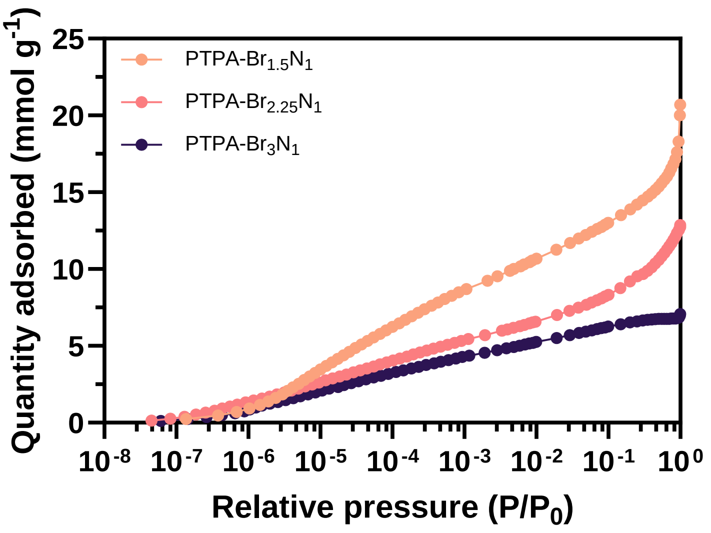

Figure S24: N_2 adsorption isotherms of the three most porous polymers (PTPA-$Br_{1.5}N_1$, PTPA-$Br_{2.25}N_1$, and PTPA-Br_3N_1) collected at 77 K and plotted with P/P_0 on a logarithmic scale to highlight the micropore filling at low relative pressures.

12 Determination of specific surface areas

Brunauer—Emmett—Teller (BET) theory [3] was used to determine the specific surface areas (S_{BET}) of the PTPA materials. The BET transformation is calculated as,

$$y = \frac{1}{Q(P_0/P - 1)}$$

where Q is the adsorbed gas quantity. A least-squares fit is performed and the following are calculated:

1. Slope, s
2. y-intercept, y_0
3. Uncertainty of the slope, $u(s)$
4. Uncertainty of the y-intercept, $u(y_0)$
5. Correlation coefficient

Subsequently, the following can be calculated,

$$A_{\mathrm{s}} = \frac{A_{\mathrm{m}} N_{\mathrm{A}}}{V_{\mathrm{m}}(s + y_0)} \times 10^{-18} m^2/nm^2$$

where A_{s} is the specific surface area (S_{BET}), A_{m} is the molecular cross-sectional area of N_2 gas in a close-packed liquid monolayer (0.162 nm^2) [4], N_{A} is the Avogadro constant and V_{m} is the molar volume of the N_2 gas.

The C term is related to the energy of adsorption of the first layer of adsorbate molecules on the adsorbent surface compared to the subsequent layers. The BET C value is calculated by,

$$C = \frac{s}{y_0} + 1$$

The quantity in the monolayer, Q_{m}, is calculated by,

$$Q_{\mathrm{m}} = \frac{1}{C y_0} = \frac{1}{s + y_0}$$

The error in A_s is calculated by,

$$u(A_s) = \frac{\sqrt{u^2(s) + u^2(y_0)}}{s + y_0}$$

Figure S25 shows plots of these transformations and calculated parameters for all PTPA materials analysed by gas sorption. Table S3 shows all parameters determined for PTPAs using BET theory.

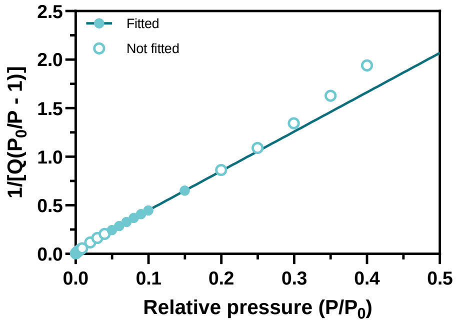

(a) PTPA-Br_1N_1

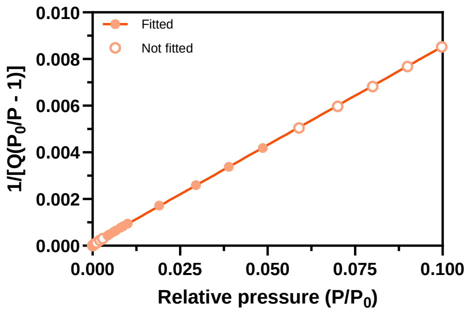

(b) PTPA-$Br_{1.5}N_1$

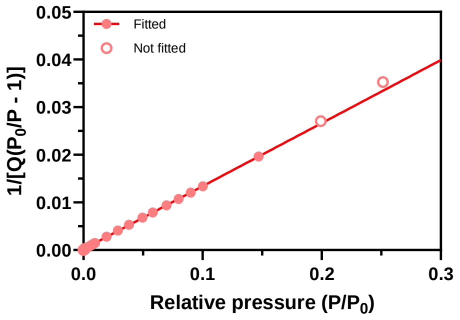

(c) PTPA-$Br_{2.25}N_1$

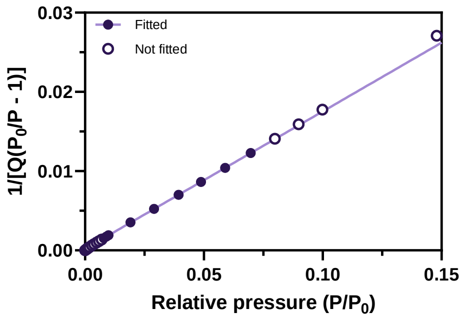

(d) PTPA-Br_3N_1

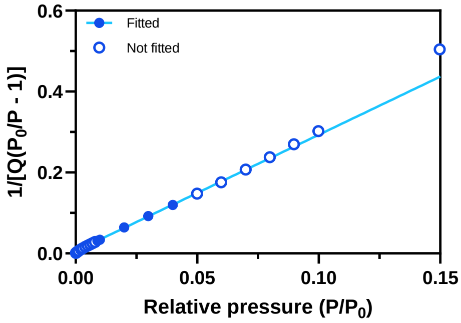

(e) PTPA-$Br_1N_{1.5}$

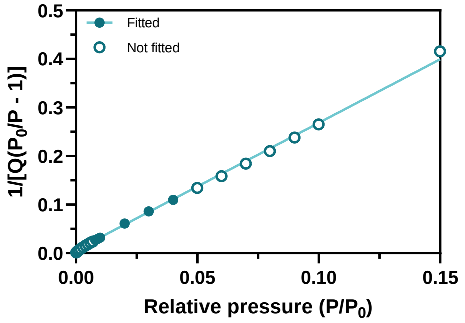

(f) PTPA-$Br_1N_{2.25}$

Figure S25: Multipoint BET plots of all PTPA samples.

Table S3: Summary of parameters determined for PTPAs using BET theory.

CMP	S_{BET} ($m^2\,g^{-1}$)	Q_m ($mmol\,g^{-1}$)	C
PTPA-Br_1N_1	23.8 ± 0.1	0.24	93
PTPA-$Br_{1.5}N_1$	1153 ± 4	11.82	926
PTPA-$Br_{2.25}N_1$	736 ± 3	7.54	751
PTPA-Br_3N_1	562 ± 1	5.76	922
PTPA-$Br_1N_{1.5}$	34 ± 1	0.35	503
PTPA-$Br_1N_{2.25}$	37 ± 1	0.38	411

13 Hydrogen sorption isotherms

13.1 Isotherms determined volumetrically at low-pressures

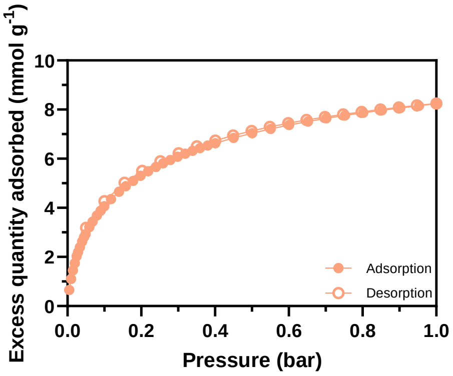

(a) Isotherm 1

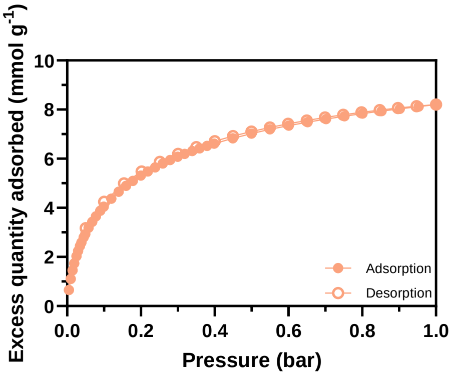

(b) Isotherm 2

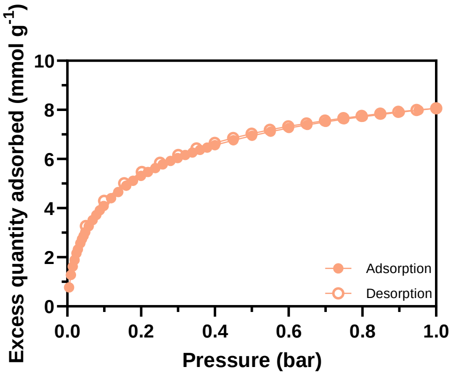

(c) Isotherm 3

Figure S26: All volumetrically determined H_2 isotherms for PTPA-$Br_{1.5}N_1$ collected at 77 K up to 1 bar. Adsorption branches are denoted by the filled symbol (-●-) and desorption branches by the empty symbol (-○-).

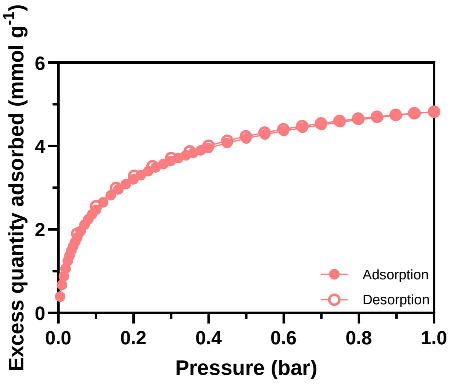

(a) Isotherm 1

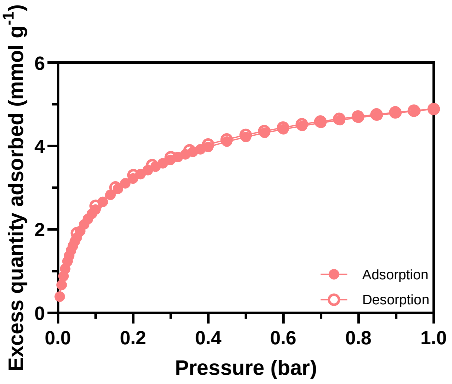

(b) Isotherm 2

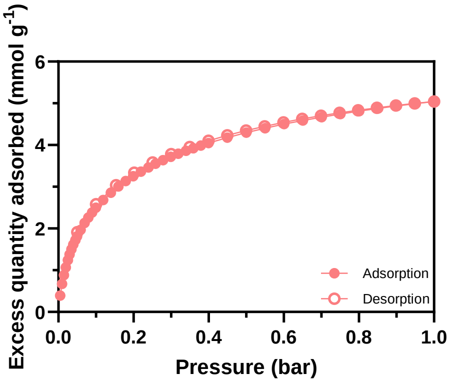

(c) Isotherm 3

Figure S27: All volumetrically determined H_2 isotherms for PTPA-$Br_{2.25}N_1$ collected at 77 K up to 1 bar. Adsorption branches are denoted by the filled symbol (-●-) and desorption branches by the empty symbol (-○-).

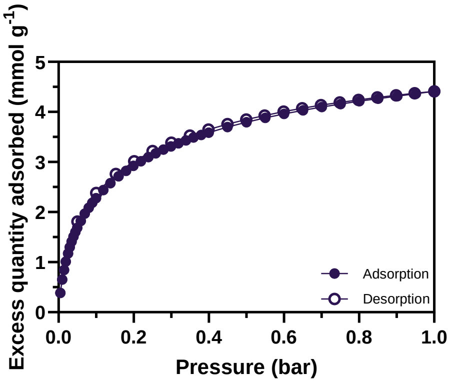

(a) Isotherm 1

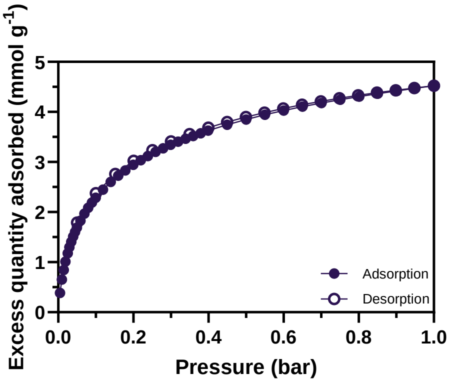

(b) Isotherm 2

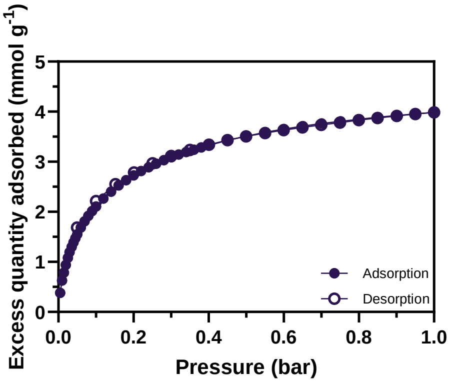

(c) Isotherm 3

Figure S28: All volumetrically determined H_2 isotherms for PTPA-Br_3N_1 collected at 77 K up to 1 bar. Adsorption branches are denoted by the filled symbol (-•-) and desorption branches by the empty symbol (-○-).

13.2 Isotherms determined gravimetrically at high-pressures

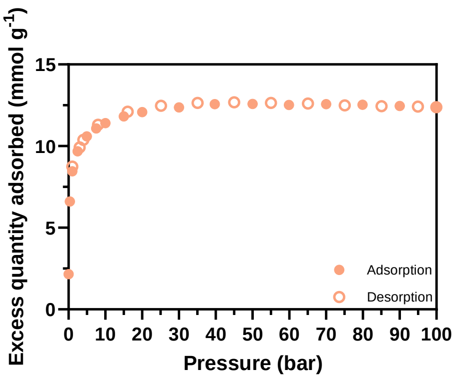

(a) Isotherm 1

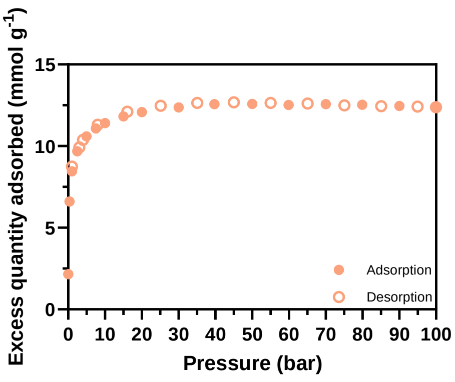

(b) Isotherm 2

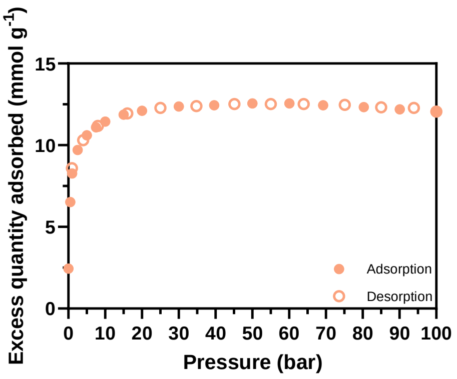

(c) Isotherm 3

Figure S29: All gravimetrically determined excess H_2 isotherms for PTPA-$Br_{1.5}N_1$ collected at 77 K up to 100 bar. Adsorption branches are denoted by the filled symbol (●) and desorption branches by the empty symbol (○).

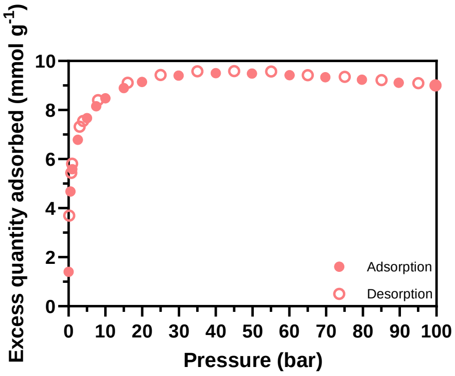

(a) Isotherm 1

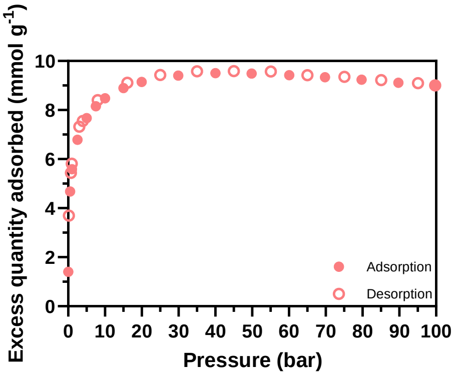

(b) Isotherm 2

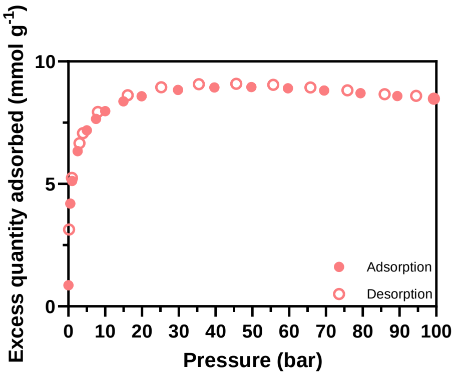

(c) Isotherm 3

Figure S30: All gravimetrically determined excess H_2 isotherms for PTPA-$Br_{2.25}N_1$ collected at 77 K up to 100 bar. Adsorption branches are denoted by the filled symbol (•) and desorption branches by the empty symbol (○).

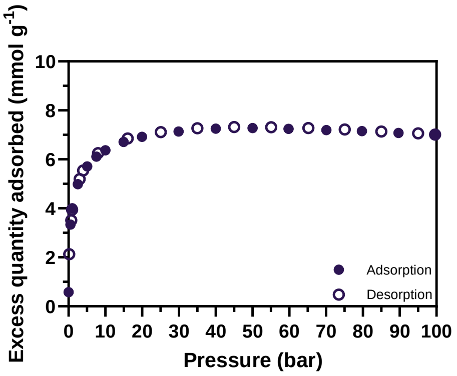

(a) Isotherm 1

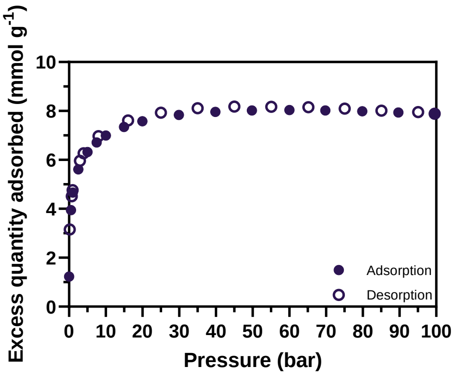

(b) Isotherm 2

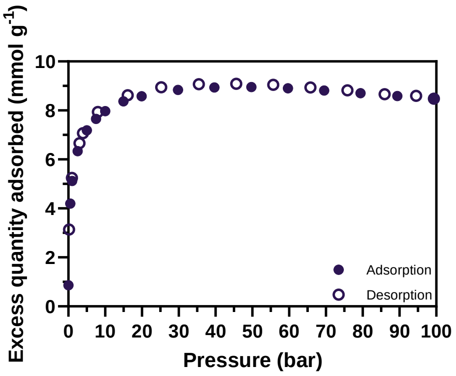

(c) Isotherm 3

Figure S31: All gravimetrically determined excess H_2 isotherms for PTPA-Br_3N_1 collected at 77 K up to 100 bar. Adsorption branches are denoted by the filled symbol (•) and desorption branches by the empty symbol (∘).

13.3 High-pressure total isotherms

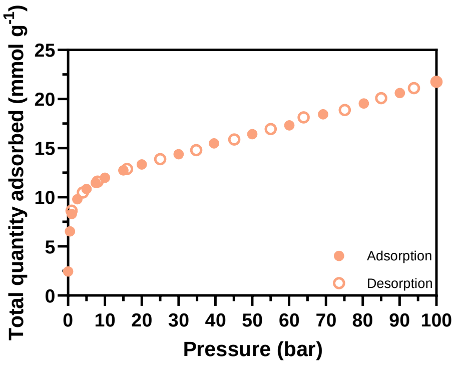

(a) Isotherm 1

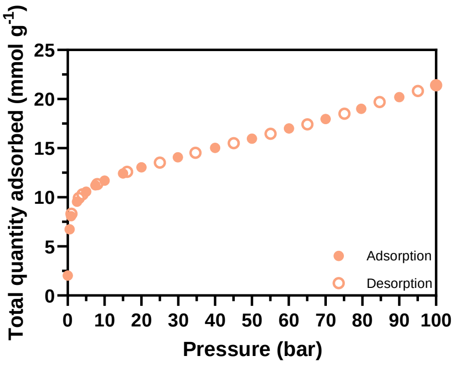

(b) Isotherm 2

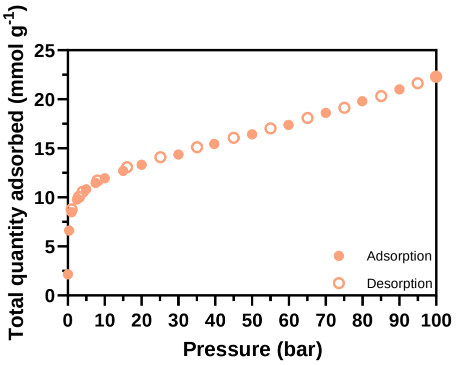

(c) Isotherm 3

Figure S32: All calculated total H_2 isotherms for PTPA-$Br_{1.5}N_1$ at 77 K up to 100 bar. Adsorption branches are denoted by the filled symbol (●) and desorption branches by the empty symbol (○).

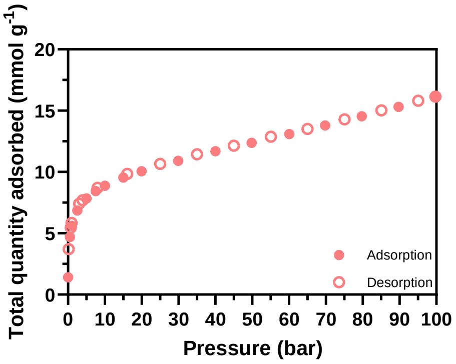

(a) Isotherm 1

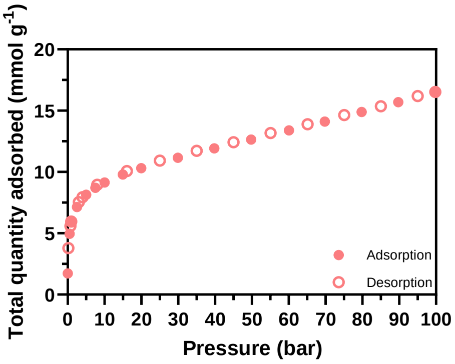

(b) Isotherm 2

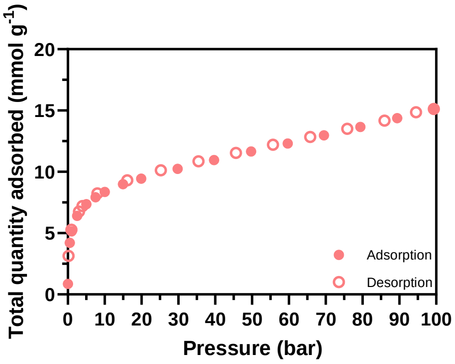

(c) Isotherm 3

Figure S33: All calculated total H_2 isotherms for PTPA-$Br_{2.25}N_1$ at 77 K up to 100 bar. Adsorption branches are denoted by the filled symbol (●) and desorption branches by the empty symbol (○).

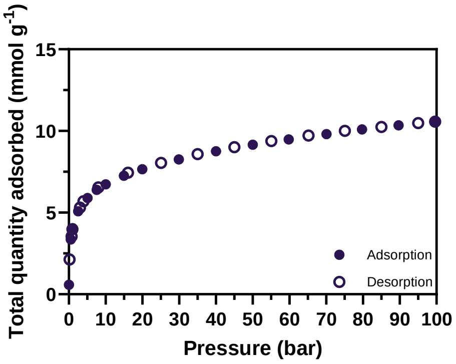

(a) Isotherm 1

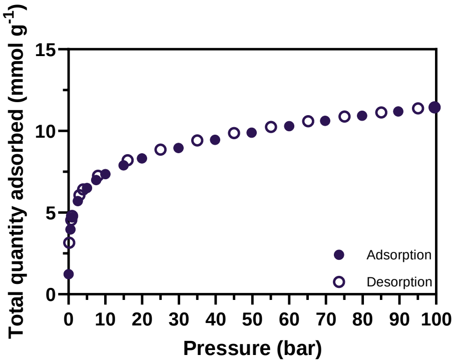

(b) Isotherm 2

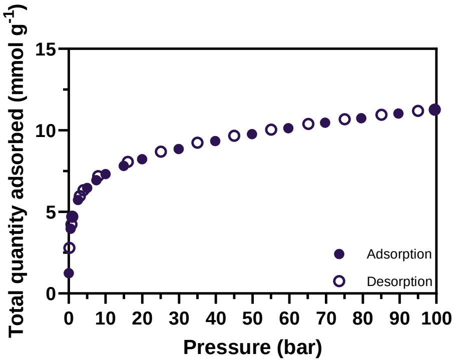

(c) Isotherm 3

Figure S34: All calculated total H_2 isotherms for PTPA-Br_3N_1 at 77 K up to 100 bar. Adsorption branches are denoted by the filled symbol (•) and desorption branches by the empty symbol (○).

13.4 High-pressure net isotherms

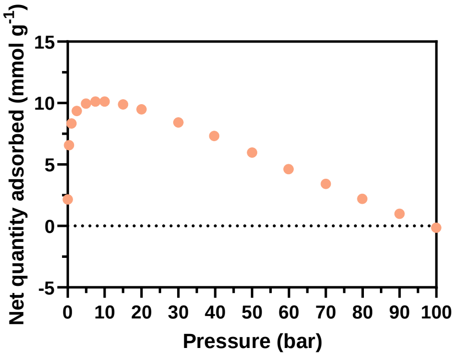

(a) Isotherm 1

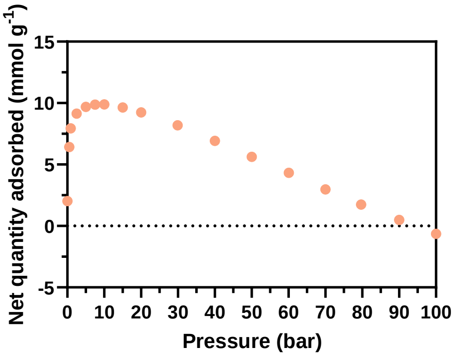

(b) Isotherm 2

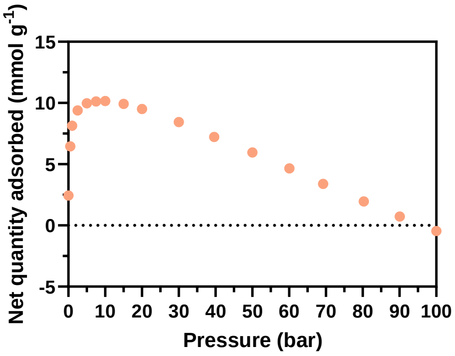

(c) Isotherm 3

Figure S35: All calculated net H_2 isotherms for PTPA-$Br_{1.5}N_1$ at 77 K up to 100 bar. Adsorption branches are denoted by the filled symbol (●).

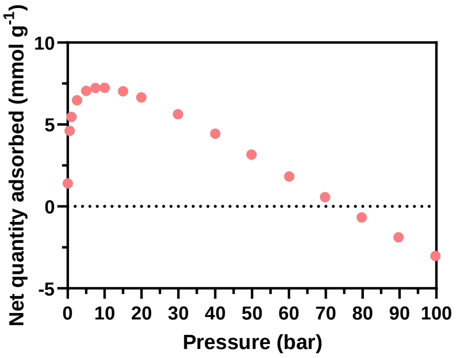

(a) Isotherm 1

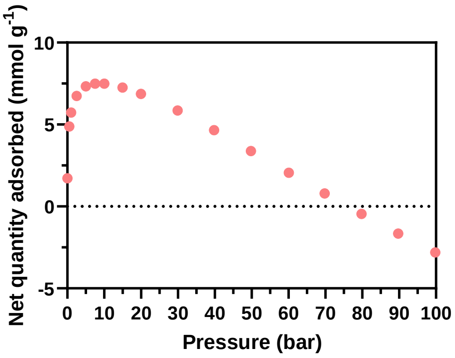

(b) Isotherm 2

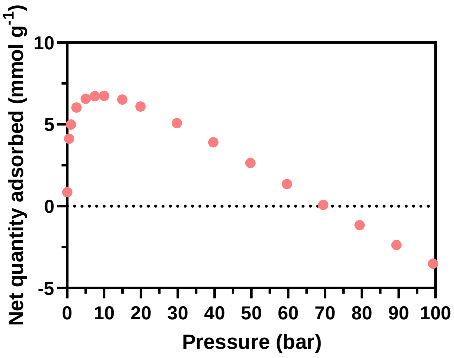

(c) Isotherm 3

Figure S36: All calculated net H_2 isotherms for PTPA-$Br_{2.25}N_1$ at 77 K up to 100 bar. Adsorption branches are denoted by the filled symbol (●).

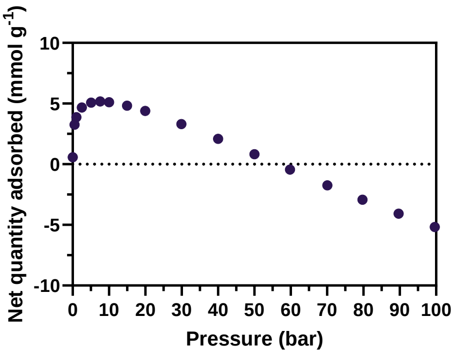

(a) Isotherm 1

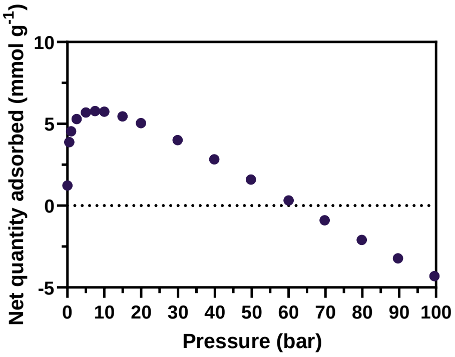

(b) Isotherm 2

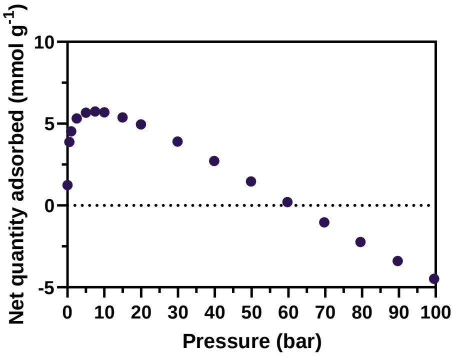

(c) Isotherm 3

Figure S37: All calculated net H_2 isotherms for PTPA-Br_3N_1 at 77 K up to 100 bar. Adsorption branches are denoted by the filled symbol (•).

14 Helium pycnometry

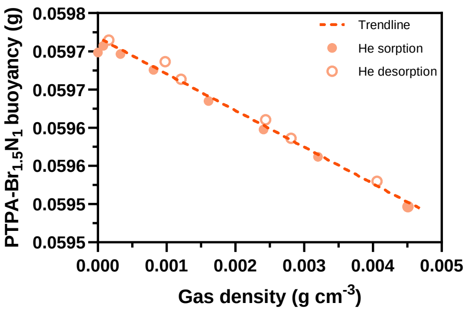

(a) PTPA-$Br_{1.5}N_1$

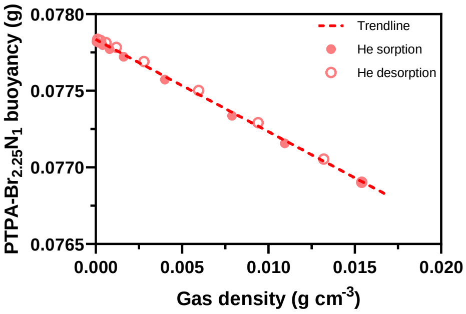

(b) PTPA-$Br_{2.25}N_1$

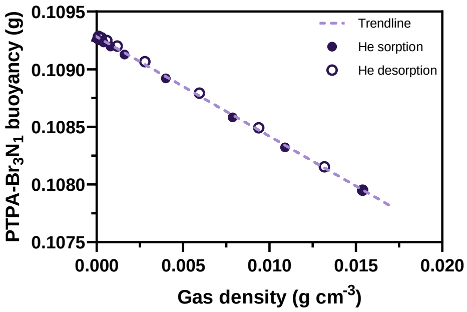

(c) PTPA-Br_3N_1

Figure S38: Sample buoyancy versus He gas density at approximately evenly spaced pressures measured at ambient temperatures (25–27 °C). Adsorption branches are denoted by the filled symbol (•) and desorption branches by the empty symbol (○). Linear fits are also shown and denoted by the dashed line (- - -).

Table S4: Summary of skeletal volumes (V_{sk}) of PTPA samples determined by He pycnometry. Uncertainties calculated from the linear regression fittings are given.

CMP	V_{sk} (cm^3)
PTPA-$Br_{1.5}N_1$	0.048 ± 0.001
PTPA-$Br_{2.25}N_1$	0.060 ± 0.001
PTPA-Br_3N_1	0.086 ± 0.001

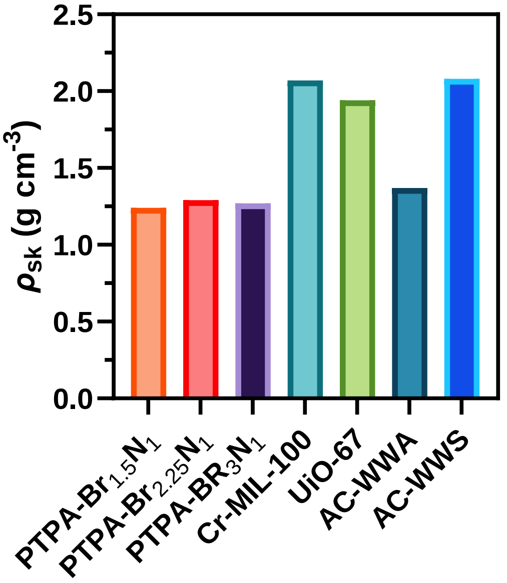

Figure S39: Skeletal densities of PTPAs, Cr-MIL-100 and UiO-67 MOFs, and AC-WWA and AC-WWS activated carbons [5, 6].

15 Fractional transient uptake plots, linear driving force fittings and calculated rates

The linear driving force fitting is given as,

$$y = y_0 + \Delta y \left[1 - e^{\frac{-(t-t_1)}{k}}\right]$$

from which k can be derived.

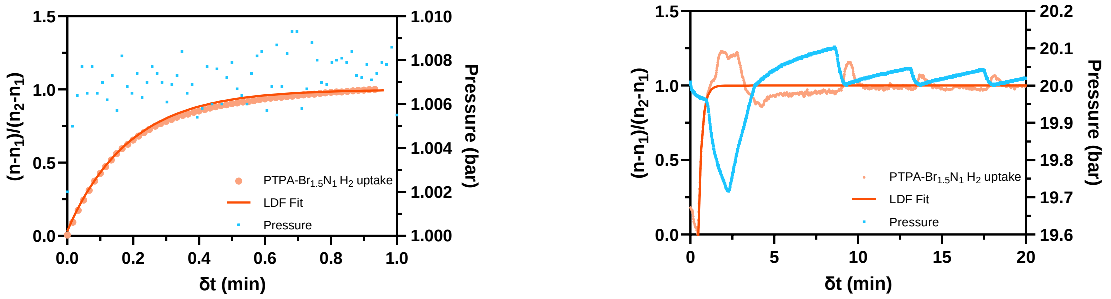

(a) Isothermal step approaching 1 bar.

(b) Isothermal step approaching 20 bar.

Figure S40: Fractional transient uptakes (●) and pressure (■) as a function of time at 77 K for the dosing step at (a) 1 bar and (b) 20 bar are shown for PTPA-Br$_{1.5}$N$_1$. The curves (–) represent the LDF fitting. $n_2 - n_1$ is the total uptake from t to t_1 in the dosing step. $n - n_1$ represents the transient uptake.

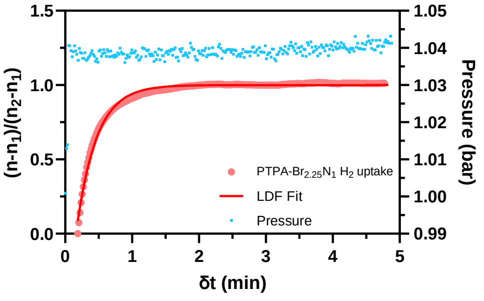

(a) Isothermal step approaching 1 bar.

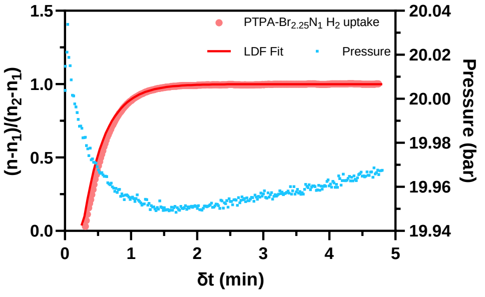

(b) Isothermal step approaching 20 bar.

Figure S41: Fractional transient uptakes (●) and pressure (■) as a function of time at 77 K for the dosing step at (a) 1 bar and (b) 20 bar are shown for PTPA-$Br_{2.25}N_1$. The curves (–) represent the LDF fitting. $n_2 - n_1$ is the total uptake from t to t_1 in the dosing step. $n - n_1$ represents the transient uptake.

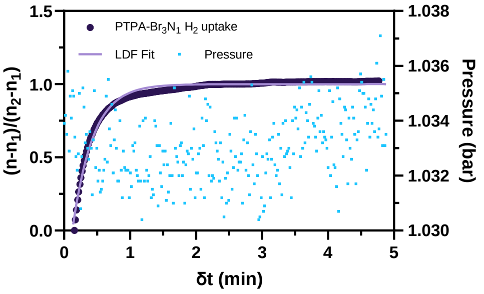

(a) Isothermal step approaching 1 bar.

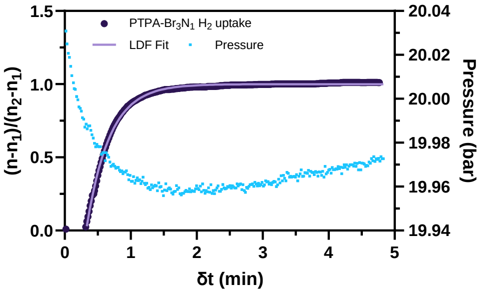

(b) Isothermal step approaching 20 bar.

Figure S42: Fractional transient uptakes (●) and pressure (■) as a function of time at 77 K for the dosing step at (a) 1 bar and (b) 20 bar are shown for PTPA-Br_3N_1. The curves (–) represent the LDF fitting. $n_2 - n_1$ is the total uptake from t to t_1 in the dosing step. $n - n_1$ represents the transient uptake.

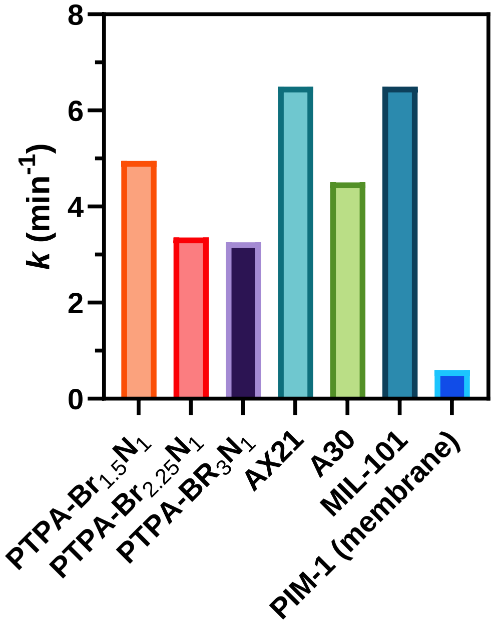

Figure S43: Mass transfer coefficient values (k) for PTPAs, activated carbons AX21 and A30, the MOF MIL-101, and the organic polymer PIM-1 in membrane form [7] calculated from data collected at comparable isothermal pressure steps (approaching 1 bar) using the LDF model [8].

References

[1] E. W. Lemmon, I. H. Bell, M. L. Huber, M. O. McLinden, NIST Standard Reference Database 23: Reference Fluid Thermodynamic and Transport Properties-REFPROP, Version 9.1, National Institute of Standards and Technology, https://www.nist.gov/srd/refprop.

[2] E. W. Lemmon, I. H. Bell, M. L. Huber, M. O. McLinden, In P.J. Linstrom, W. Mallard, editors, *NIST Chemistry WebBook, NIST Standard Reference Database Number 69.* National Institute of Standards and Technology, Gaithersburg MD, 20899, USA, **1998**.

[3] S. Brunauer, P. H. Emmett, E. Teller, *J. Am. Chem. Soc.* **1938**, *60*, 2 309.

[4] P. H. Emmett, S. Brunauer, *J. Am. Chem. Soc.* **1937**, *59*, 8 1553.

[5] G. E. Decker, E. D. Bloch, *ACS Appl. Mater. Interfaces* **2021**, *13*, 44 51925.

[6] M. E. Ergün, S. Bulbul, *Int. Adv. Res. Eng. J.* **2022**, *6*, 3 167.

[7] M. Tian, S. Rochat, K. Polak-Kraśna, L. T. Holyfield, A. D. Burrows, C. R. Bowen, T. J. Mays, *Adsorption* **2019**, *25*, 4 889.

[8] E. Glueckauf, *Trans. Faraday Soc.* **1955**, *51*, 0 1540.
